# Bibliometric and visualization analysis in the field of epigenetics and glioma (2009–2024)

**DOI:** 10.3389/fonc.2024.1431636

**Published:** 2024-10-29

**Authors:** Yijun Zeng, Ge Tao, Yong Zeng, Jihong He, Hui Cao, Lushun Zhang

**Affiliations:** ^1^ Department of Neurosurgery, The Third Affiliated Hospital of Chengdu Medical College, Chengdu Pidu District People’s Hospital, Chengdu, China; ^2^ School of Clinical Medicine, Chengdu Medical College, Chengdu, China; ^3^ Development and Regeneration Key Laboratory of Sichuan Province, Institute of Neuroscience, Department of Pathology and Pathophysiology, Chengdu Medical College, Chengdu, China

**Keywords:** glioma, epigenetics, therapeutic strategies, DNA methylation, TMZ, CiteSpace, VOSviewer

## Abstract

**Introduction:**

Glioma represents the most prevalent primary malignant tumor in the central nervous system, a deeper understanding of the underlying molecular mechanisms driving glioma is imperative for guiding future treatment strategies. Emerging evidence has implicated a close relationship between glioma development and epigenetic regulation. However, there remains a significant lack of comprehensive summaries in this domain. This study aims to analyze epigenetic publications pertaining to gliomas from 2009 to 2024 using bibliometric methods, consolidate the extant research, and delineate future prospects for investigation in this critical area.

**Methods:**

For the purpose of this study, publications spanning the years 2009 to 2024 were extracted from the esteemed Web of Science Core Collection (WoSCC) database. Utilizing advanced visualization tools such as CiteSpace and VOSviewer, comprehensive data pertaining to various aspects including countries, authors, author co-citations, countries/regions, institutions, journals, cited literature, and keywords were systematically visualized and analyzed.

**Results:**

A thorough analysis was conducted on a comprehensive dataset consisting of 858 publications, which unveiled a discernible trend of steady annual growth in research output within this specific field. The nations of the United States, China, and Germany emerged as the foremost contributors to this research domain. It is noteworthy that von Deimling A and the Helmholtz Association were distinguished as prominent authors and institutions, respectively, in this corpus of literature. A rigorous keyword search and subsequent co-occurrence analysis were executed, ultimately leading to the identification of seven distinct clusters: “epigenetic regulation”, “DNA repair”, “DNA methylation”, “brain tumors”, “diffuse midline glioma (DMG)”, “U-87 MG” and “epigenomics”. Furthermore, an intricate cluster analysis revealed that the primary foci of research within this field were centered around the exploration of glioma pathogenesis and the development of corresponding treatment strategies.

**Conclusion:**

This article underscores the prevailing trends and hotspots in glioma epigenetics, offering invaluable insights that can guide future research endeavors. The investigation of epigenetic mechanisms primarily centers on DNA modification, non-coding RNAs (ncRNAs), and histone modification. Furthermore, the pursuit of overcoming temozolomide (TMZ) resistance and the exploration of diverse emerging therapeutic strategies have emerged as pivotal avenues for future research within the field of glioma epigenetics.

## 1 Introduction

Gliomas, which typically arise from neuroglial stem or progenitor cells, are commonly categorized into three distinct subtypes based on their histological characteristics: astrocytic tumors, oligodendroglial tumors, and ventriculomembranous tumors (1). According to the database maintained by the International Agency for Research on Cancer, brain and central nervous system cancers presently constitute 3% of all cancer-related deaths (2). Furthermore, projections suggest a potential for this figure to nearly triple by the year 2040 (3). Malignant gliomas represent the second leading cause of cancer-related deaths among individuals under the age of 35 (4). Between 2014 and 2018 in the United States, an average of 16,606 deaths per year were attributed to malignant brain and other central nervous system tumors, resulting in a mortality rate of 4.43 deaths per 100,000 individuals (5). Gliomas constitute the most prevalent primary malignant brain tumors, accounting for approximately 30% of all brain tumor cases, and are among the most lethal forms of this disease (6). According to the latest definition by the World Health Organization (WHO), adult gliomas are primarily classified as WHO grade II to IV tumors (7). Among these glioma classifications, glioblastoma (GBM, WHO grade IV) is considered one of the most lethal types (8). For GBM, the Stupp regimen has been reported to result in a median survival of approximately 14.6 months, a median progression-free survival of 6.9 months, and a two-year overall survival rate of 26.5% (9–11). Gliomas present significant treatment challenges owing to their poor prognosis, high recurrence rate, and considerable mortality rate, thereby posing a severe threat to the health of patients (6).

Current treatments for gliomas primarily include surgery, chemotherapy and radiotherapy (12). However, the notable heterogeneity exhibited by glioma cells plays a pivotal role in their heightened resistance to both chemotherapy and radiotherapy (13). However, the substantial heterogeneity observed among glioma cells contributes significantly to their pronounced resistance to both chemotherapy and radiotherapy (14). The emergence of increasing resistance to TMZ, coupled with the drug’s ineffective distribution across the blood-brain barrier (BBB), has posed significant challenges in the treatment of gliomas (15). Despite the advent of numerous emerging therapeutic strategies, none have ushered in fundamental alterations in the management of glioma patients (16). Recent studies in molecular pathology have uncovered a significant association between the development of gliomas and various epigenetic phenomena. Mutations in genes regulated by epigenetic mechanisms have been pinpointed as pivotal factors in the formation of distinct glioma subtypes. Additionally, epigenetic alterations hold promise as valuable biomarkers, offering novel avenues for classifying gliomas and informing treatment decisions (17). Epigenetics refers to heritable changes in cellular phenotypes that occur without alterations in the DNA sequence (18). Recent research has pinpointed several key components of epigenetic regulatory mechanisms in gliomas, encompassing DNA methylation, dysregulation of ncRNAs, chromatin remodeling, and abnormalities in histone modifications (19). Epigenetic factors play a pivotal role in the diagnosis and treatment of diseases (20). Current research on glioma epigenetics centers around several pivotal areas: DNA methylation, integrated genomic analysis, epigenetic silencing, histone posttranslational modifications, and ncRNAs. The pathogenesis of gliomas is influenced by a complex interplay of genetic and environmental factors, posing significant challenges for treatment and diagnosis. By elucidating the reversibility of epigenetic changes, researchers aim to unravel the underlying causes of glioma development, potentially paving the way for novel therapeutic strategies. However, there remains a paucity of statistical data on the research frontiers and emerging trends in this field.

Bibliometrics is a discipline that employs quantitative methods to systematically organize and analyze information in the field of scientific research. It involves categorizing information based on various variables, including keywords, authors, and institutions. This categorization enables the rapid identification of research trends and focal points within a specific field of study. By utilizing mathematical and statistical techniques, bibliometrics provides researchers with a comprehensive and systematic understanding of the structure and development of scientific knowledge (21). Therefore, we adopted a systematic bibliometric approach to visually present and analyze the research trends and hotspots in the field of glioma epigenetics over the past 15 years.

## 2 Materials and methods

### 2.1 Data extraction

**Figure 1:**
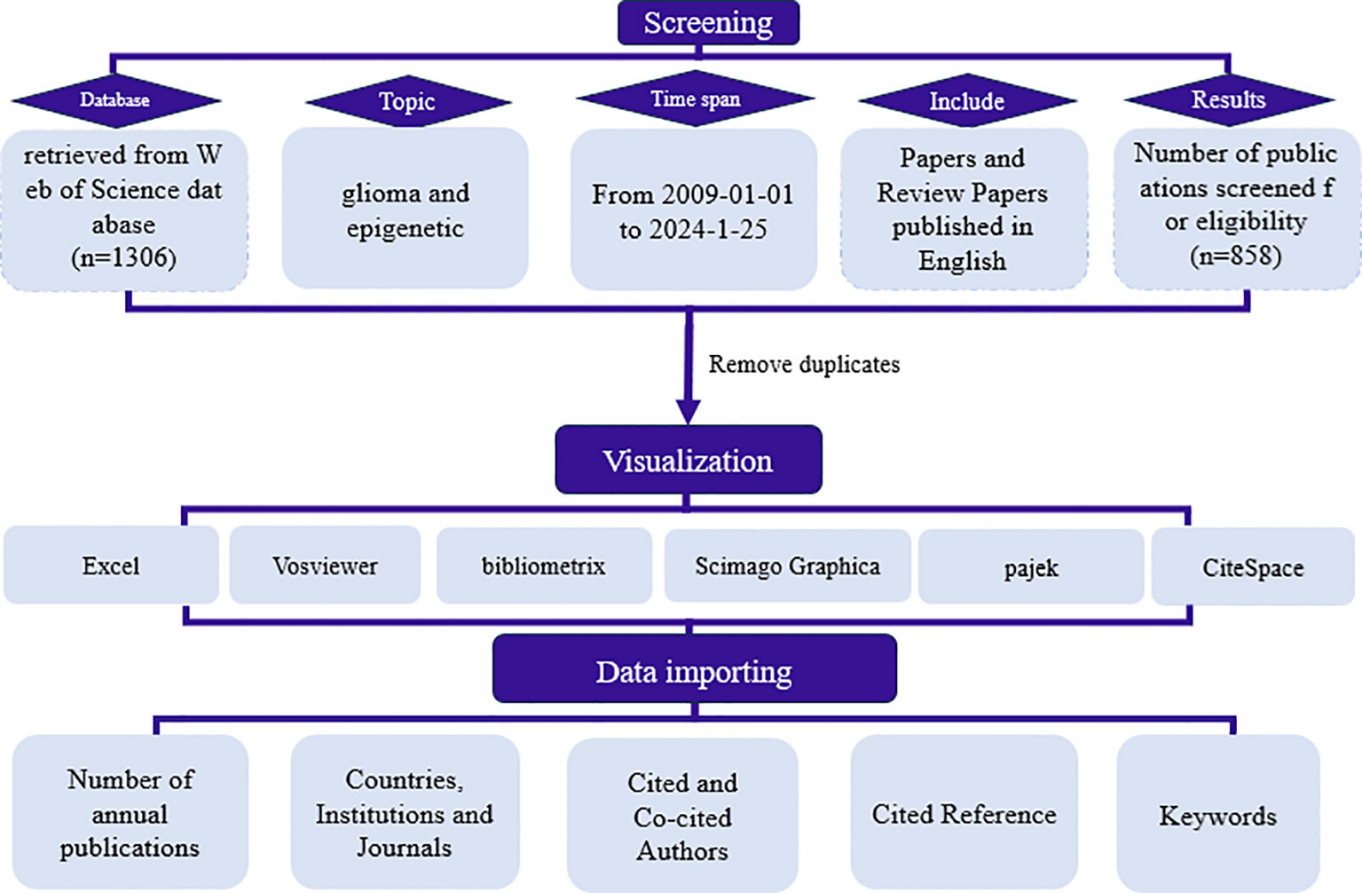
Flow chart of the literature selection process.

On January 25, 2024, a systematic literature search was conducted utilizing the WoSCC database to identify studies on glioma epigenetics published between the years 2009 and 2024. The search criteria were restricted to English-language articles, with a specific focus on glioma and epigenetics across all relevant fields to ensure comprehensive coverage. This encompassed both research articles and reviews. Initially, a total of 1,306 articles were retrieved and subjected to screening based on their titles, keywords, abstracts, and full texts. After the exclusion of irrelevant papers and the removal of duplicates, a final selection of 858 articles was made for further analysis. The selection process is depicted in **Figure 1**.

### 2.2 Data analysis and analysis tools

We conducted bibliometric and visualization analyses of the literature on glioma epigenetics using CiteSpace (version 6.2. R4), VOSviewer (version 1.6.11), Scimago Graphica, Pajeck32 portable (version 5.18), and the “bibliometrix” package in RStudio with R software (version 4.3.2). The data were statistically analyzed using Microsoft Excel 2019. The analysis covered three main aspects: 1. Statistical analysis, which involved annual publication trends, countries/regions of publication, institutions, authors, cited authors and journals. 2. Co-citation cluster analysis of references to identify key research themes and track research trends. 3. Keyword analysis to identify current research hotspots. CiteSpace, a bibliometric software package developed by Prof. Chen, utilizes co-occurrence network mapping to visualize relationships within the literature (22). In CiteSpace, node size reflects co-occurrence frequency, node connections represent relationships, line strength indicates relationship strength, and node connection color signifies different years. Purple circles outside nodes indicate centrality ≥ 0.1, highlighting nodes that play a mediating role in the cooperative network. VOSviewer, another bibliometric software developed by Leiden University, offers user-friendly operation and produces concise graphs. The results were presented in both general views and heatmap formats, which were utilized in this study for analyzing cited authors and keywords.

## 3 Results

### 3.1 Analysis of annual publications

**Figure 2:**
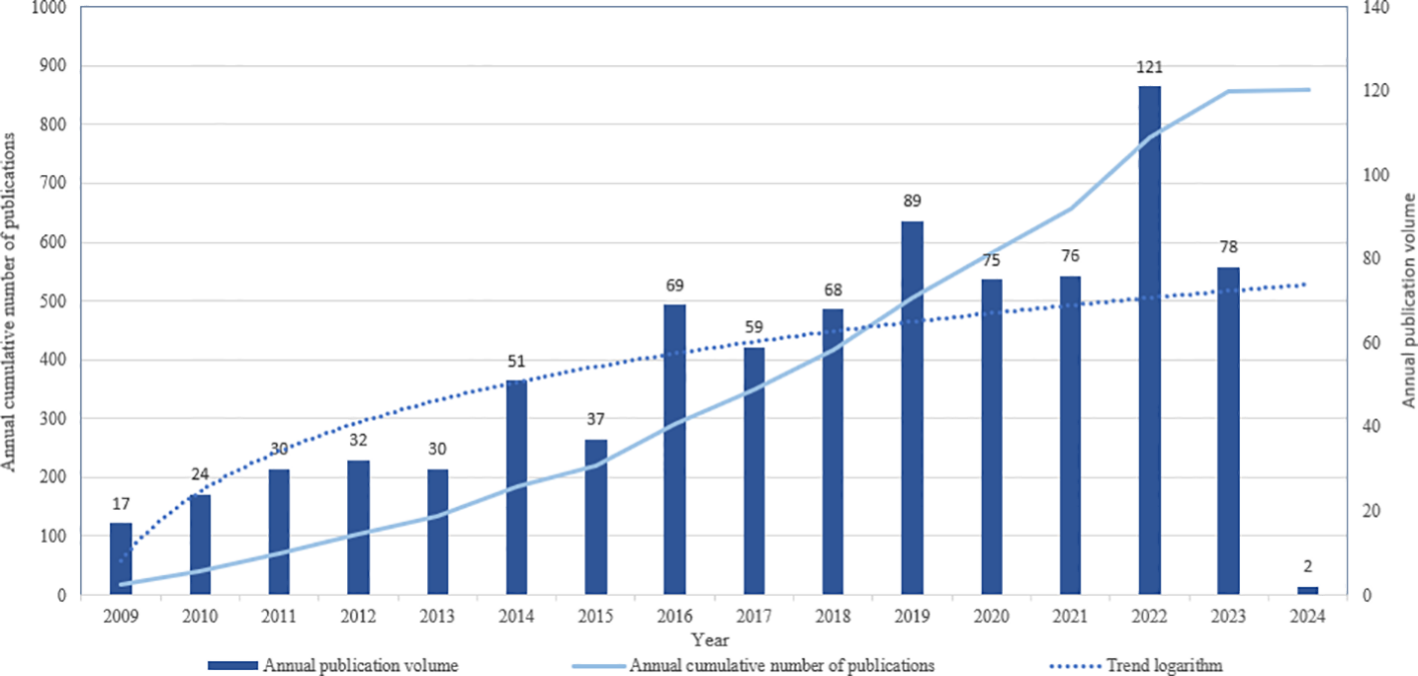
Annual number of publications in the relevant literature from 2009 to January 2024.

A comprehensive search was conducted to identify English-language papers on glioma and epigenetics, encompassing both articles and reviews, published between January 1, 2009, and February 1, 2024. This search yielded a total of 858 papers. Statistical analysis of these papers revealed significant changes in publication volumes over time. Specifically, from 2009 to 2013, there was a slow but steady increase in the number of publications in this field. However, a notable rise in publication output was observed since 2014, with a peak occurring in 2022. The logarithmic trend line clearly illustrates this marked increase, indicating a growing interest and research focus on glioma and epigenetics. Consequently, the surge in the number of published papers underscores the current importance and prominence of glioma and epigenetics as a research area (**Figure 2**).

### 3.2 Distribution of countries/regions and institutions

**Figure 3:**
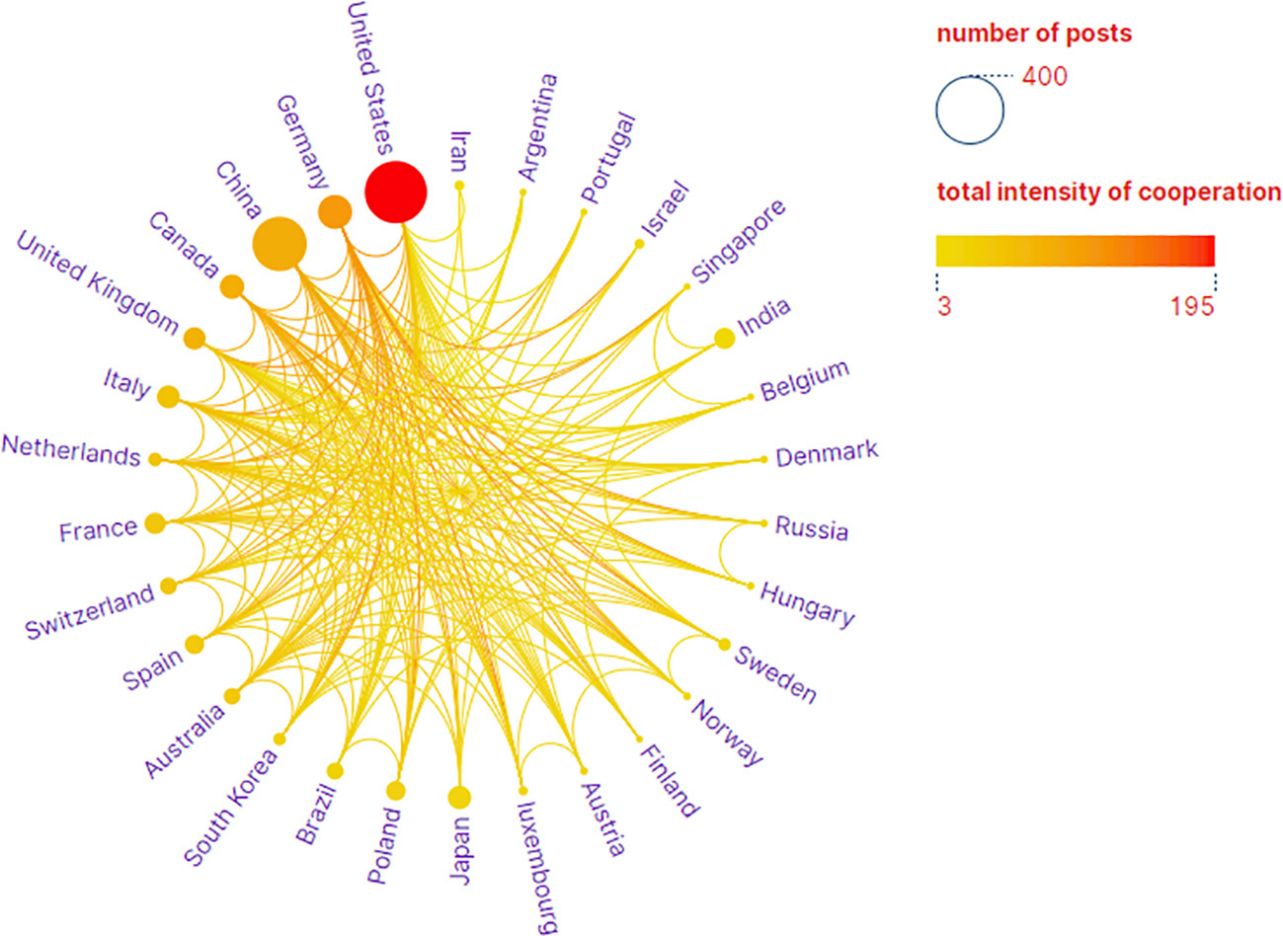
National Partnership Network. Visual map showing the country cooperation network, with circles representing a country/region and the color and size of the circles being proportional to the number of publications.

**Figure 4:**
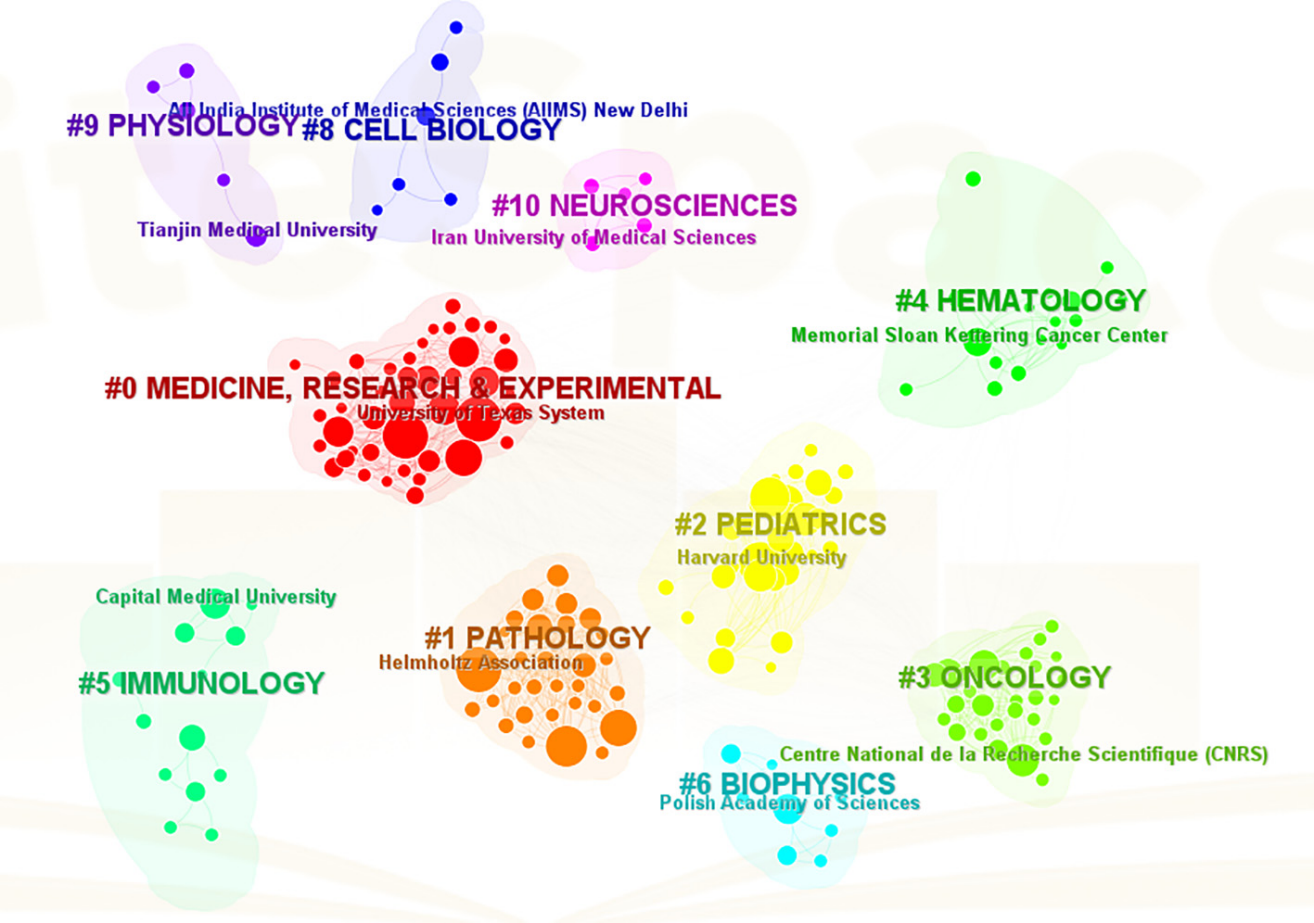
A visual map of thematic clusters of disciplines to which the institution belongs. The clusters are categorized into clusters #0-10 by different colors, and the clustered subjects are displayed on the corresponding cluster blocks.

The analysis conducted comprehensively identified a total of 58 countries/regions and 316 institutions actively engaged in epigenetic studies of gliomas. The findings revealed that the United States contributed the largest number of publications, with 305 publications accounting for 34.00% of the total. China followed closely, contributing 226 publications, which represented 25.19% of the overall publications. Germany and Canada also demonstrated significant contributions, with 92 publications (10.26%) and 46 publications (5.13%), respectively. These results highlight the prominent role played by these countries in advancing research on the epigenetic aspects of gliomas. (**Table 1**). The papers from United States received the highest number of citations (16,616 citations). **Figure 3** illustrates collaborations among the top 30 countries/regions in glioma epigenetics research, based on publication numbers. The United States dominates the field with a centrality value of 0.3, while Germany exhibits significant influence with a centrality value of 0.14. This underscores their prominent roles in advancing glioma epigenetics research. **Table 2** presents the top 10 institutions in glioma epigenetics research by publication count. The Helmholtz Association leads with 42 publications, followed closely by the University of Texas System (n=41) and the University of California System (n=40). A significant majority of these leading institutions are located in the United States (60%) and Germany (30%). Notably, the University of California System holds the highest centrality value of 0.28, indicating its substantial influence in the field. The University of Texas System follows with a centrality value of 0.13. Both institutions are based in the United States. **Figure 4** categorizes these institutions into ten clusters based on their disciplinary focus. The clusters emphasize “medical research and experimentation”, “pathology” and “pediatrics”, highlighting the key areas of research within glioma epigenetics.

**Table 1:** Top 10 countries/regions for related publications from 2009 to 2024.

Rank	Counts	Country	Citations	Centrality
1	305	United States	16,616	0.3
2	226	China	5,756	0.09
3	92	Germany	5,054	0.14
4	46	Canada	3,069	0.04
5	44	Japan	942	0
6	41	Italy	1,347	0.12
7	40	England	2,664	0.1
8	36	France	1,312	0.02
9	36	India	664	0
10	31	Poland	2,023	0

**Table 2:** Top 10 most active organizations and their countries of affiliation.

Rank	Institution	Documents	Centrality	Country
1	Helmholtz Association	42	0.06	Germany
2	University of Texas System	41	0.13	United States
3	University of California System	40	0.28	United States
4	Harvard University	38	0.09	United States
5	German Cancer Research Center (DKFZ)	36	0.06	Germany
6	Northwestern University	29	0.01	United States
7	Feinberg School of Medicine	27	0.01	United States
8	Ruprecht Karls University Heidelberg	27	0.04	Germany
9	UTMD Anderson Cancer Center	27	0.05	United States
10	Centre National de la Recherche Scientifique (CNRS)	21	0.01	France

### 3.3 Analysis of authors and author co-citations

**Figure 5:**
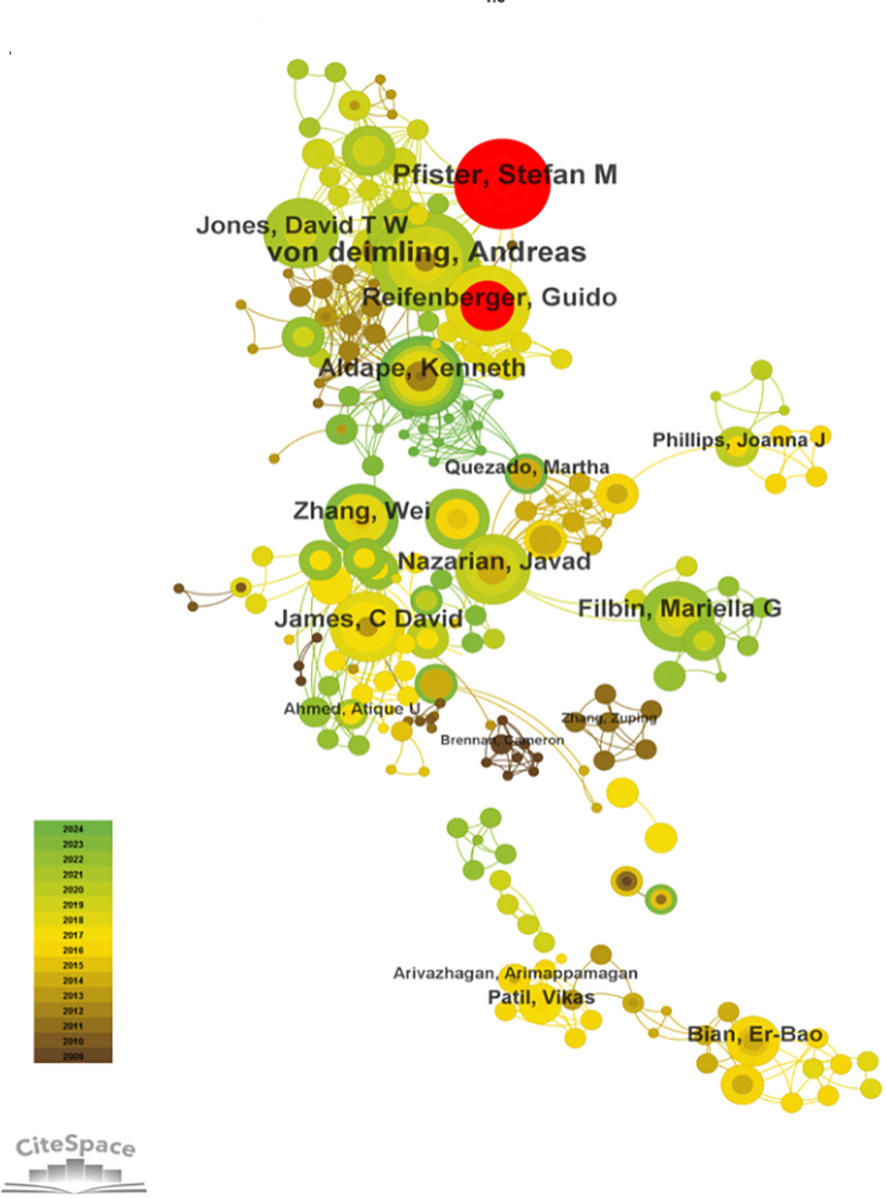
Collaborative network of authors involved in glioma and epigenetic studies.

**Figure 6:**
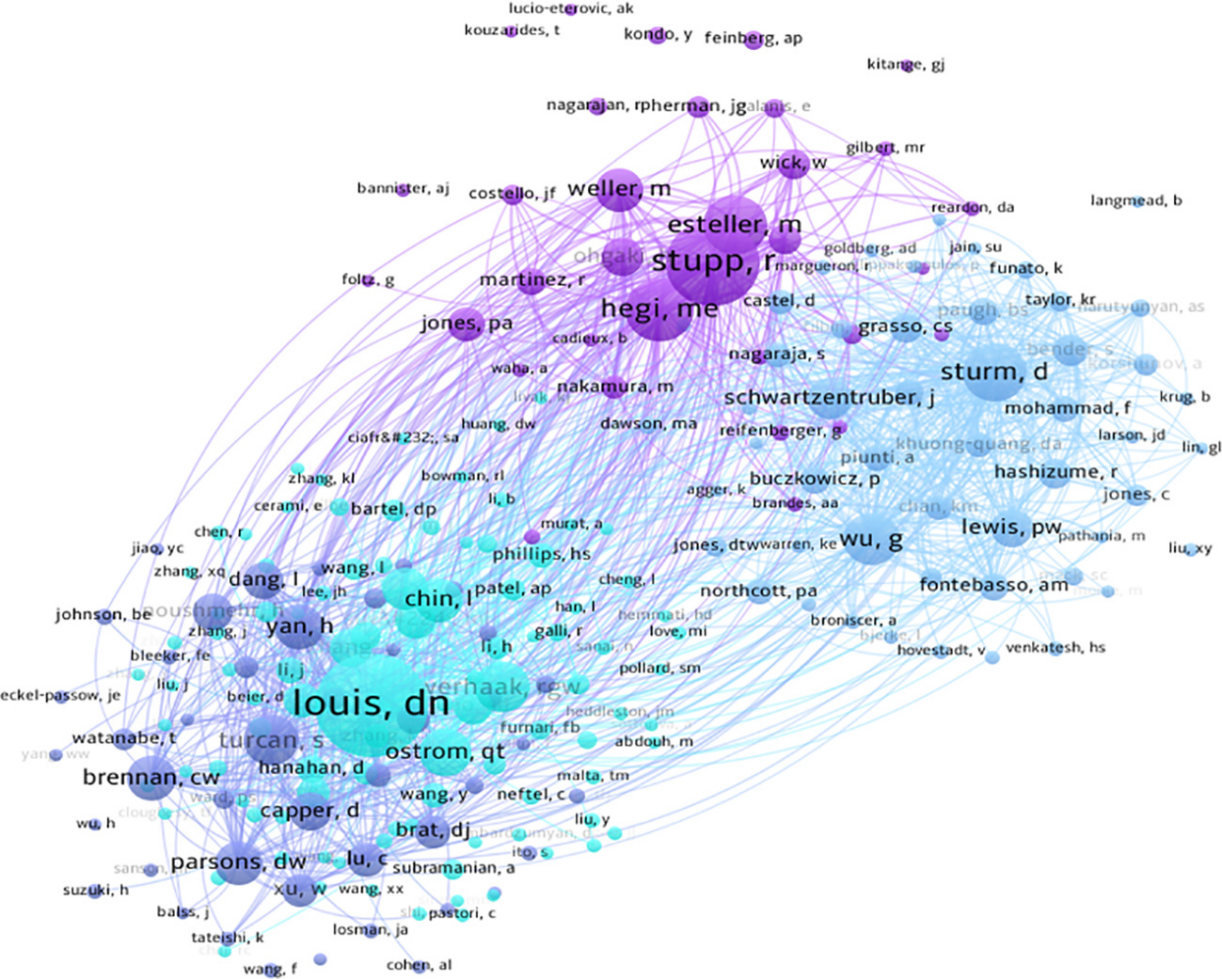
Visualizing collaborative networks of co-cited authors.

Using CiteSpace for author co-occurrence network analysis (**Figure 5**), we found that 279 researchers contributed to the relevant literature publications in glioma epigenetics. **Table 3** ranks the top 10 authors by their publication output, with von Deimling A leading with 10 publications. Pfister SM follows with 9 publications, and Aldape K and Reifenberger G each have 8 publications. Among these leading authors, Aldape K has the highest centrality at 0.07, indicating significant influence in the research community. Zhang W, James CD, and Nazarian J each have a centrality of 0.03. Author co-citation analysis was conducted to identify citation relationships between authors whose work is cited together by a third author. This method provides valuable insights into major academic researchers and their specific areas of expertise in glioma epigenetics (23). Louis DN is the most cited author with 276 citations, followed by Stupp R with 179 citations, and Hegi ME with 139 citations. **Figure 6** displays a visual network diagram illustrating the relationships between co-cited authors.

**Table 3:** The top 10 authors with the most publications.

Rank	Authors	Counts	Centrality
1	Von Deimling A	10	0.01
2	Pfister SM	9	0
3	Aldape K	8	0.07
4	Reifenberger G	8	0.01
5	Zhang W	7	0.06
6	James CD	7	0.03
7	Nazarian J	7	0.03
8	Filbin MG	7	0.03
9	Jones DTW	7	0
10	Filbin MG	7	0.01

### 3.4 Analysis of journals and cited journals for publications on glioma epigenetics

**Figure 7:**
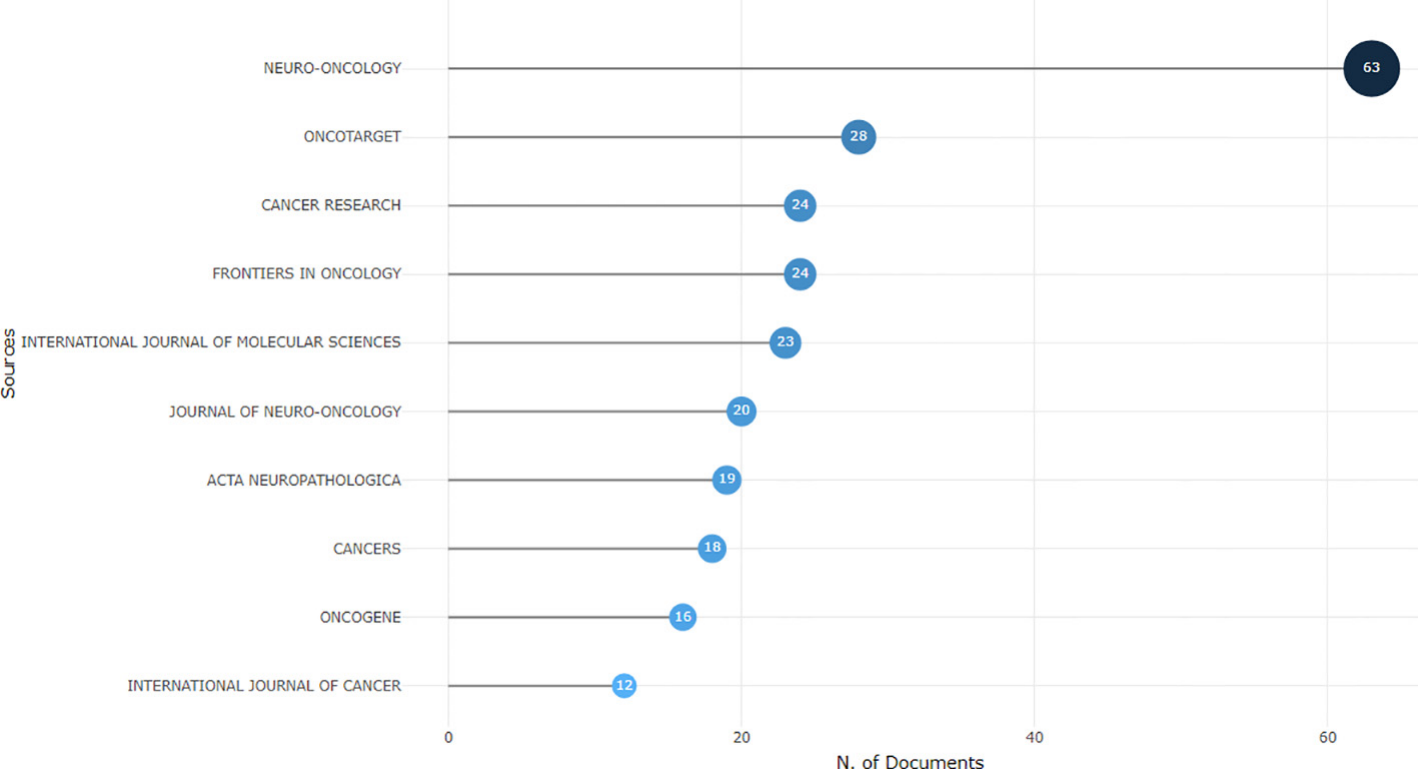
Visual presentation of the top ten journals belonging to the glioma and epigenetic research literature.

**Figure 8:**
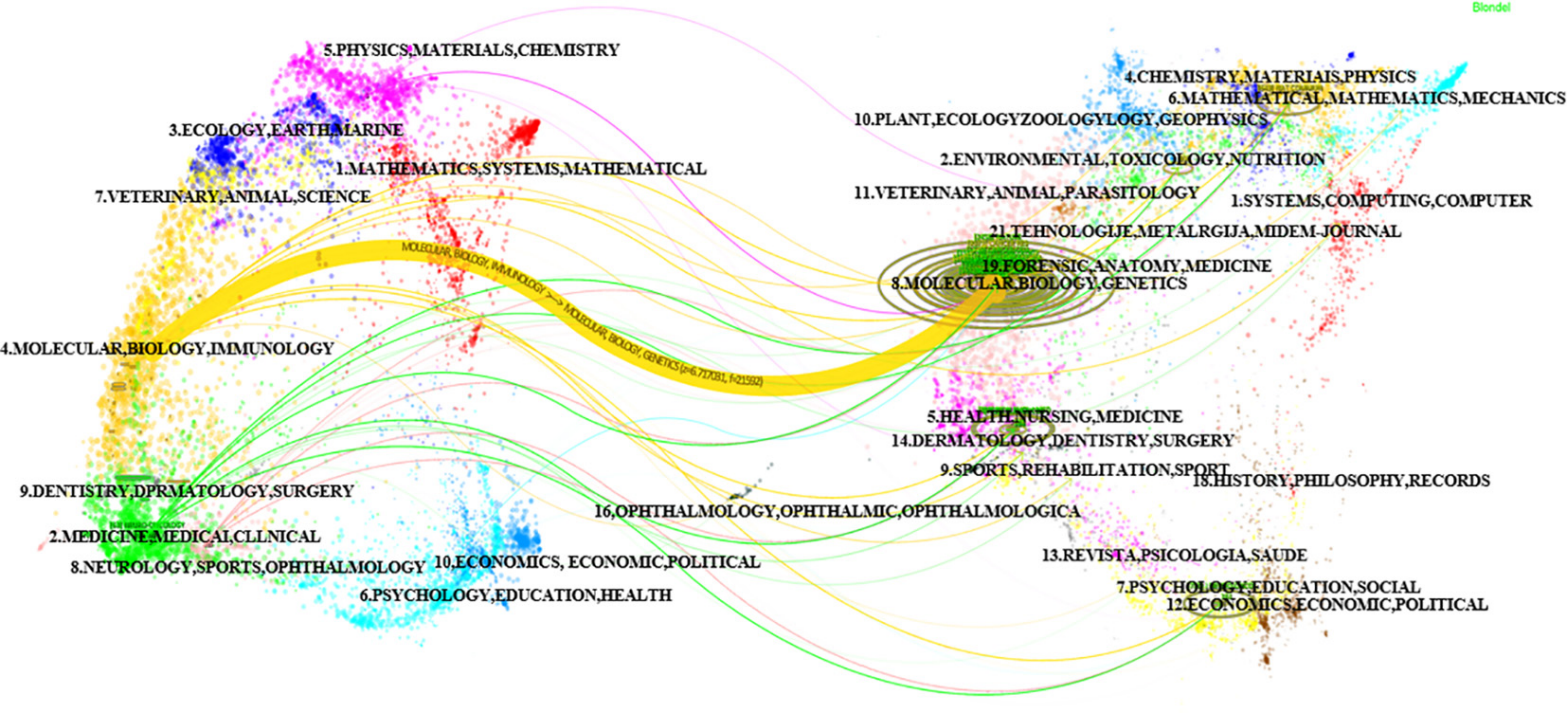
Double image overlay of journals.

Using the bibliometric online analysis platform bibliometrix, we conducted a comprehensive analysis of the literature sourced from various journals. Our analysis revealed a total of 316 journals that have published articles in the field of glioma epigenetics. **Figure 7** presents a visual mapping of the top ten journals by the number of articles published. Neuro-Oncology emerged as the predominant journal in this field, with a total of 63 articles. This finding suggests that Neuro-Oncology is a key platform for disseminating research related to glioma epigenetics. Following Neuro-Oncology, Oncotarget ranks second with 28 articles, and Cancer Research ranks third with 24 articles. These journals also contribute significantly to the publication of research in this field. The relatively uniform distribution of articles across the other journals indicates the diversity of research in glioma epigenetics. Researchers are contributing their work to a wide range of publications, highlighting the multidisciplinary nature of this field. Overall, the analysis of journal publications demonstrates the prominence of Neuro-Oncology as the leading journal in glioma epigenetics research and the diverse range of journals in which researchers publish their work. This diversity reflects the broad scope and interdisciplinary nature of research in this field. **Figure 8** presents the journal dual-map overlays, which illustrate the positioning of research on a specific topic in relation to major research disciplines. Each point on the map represents a journal. The citation map is displayed on the left, the cited map is shown on the right, and the connecting lines in the middle represent citation paths. These linking trajectories highlight the interconnections between different disciplines within the field. The colors on the map indicate clusters identified by the Blondel clustering algorithm. On the left map, the width of the ellipses correlates with the number of publications, while the length represents the number of authors. The findings reveal that publications in the Molecular Biology and Immunology field (yellow trajectory) are significantly influenced by research in Molecular Biology and Genetics (z=6.72, f=21592). **Table 4** presents the top 10 cited journals based on citation frequency, 2022 impact factor (IF) and centrality. Nature has the highest citation frequency and impact factor, establishing it as the most influential journal in the field.

**Table 4:** Top 10 most cited journals.

Rank	Sources	TC	IF 2022	Centrality
1	Nature	2,458	64.8	0.01
2	Cancer Research	2,073	11.2	0.01
3	Cancer Cell	1,729	50.3	0.01
4	Cell	1,703	64.5	0.01
5	Neuro-Oncology	1,517	15.9	0.01
6	Science	1,292	56.9	0
7	Proceedings of The National Academy of Sciences of The United States of America	1,289	11.1	0.01
8	Acta Neuropathologica	1,180	12.7	0.01
9	Oncogene	1,090	8	0
10	Plos One	1,014	3.7	0.01

### 3.5 Citation analysis

**Figure 9:**
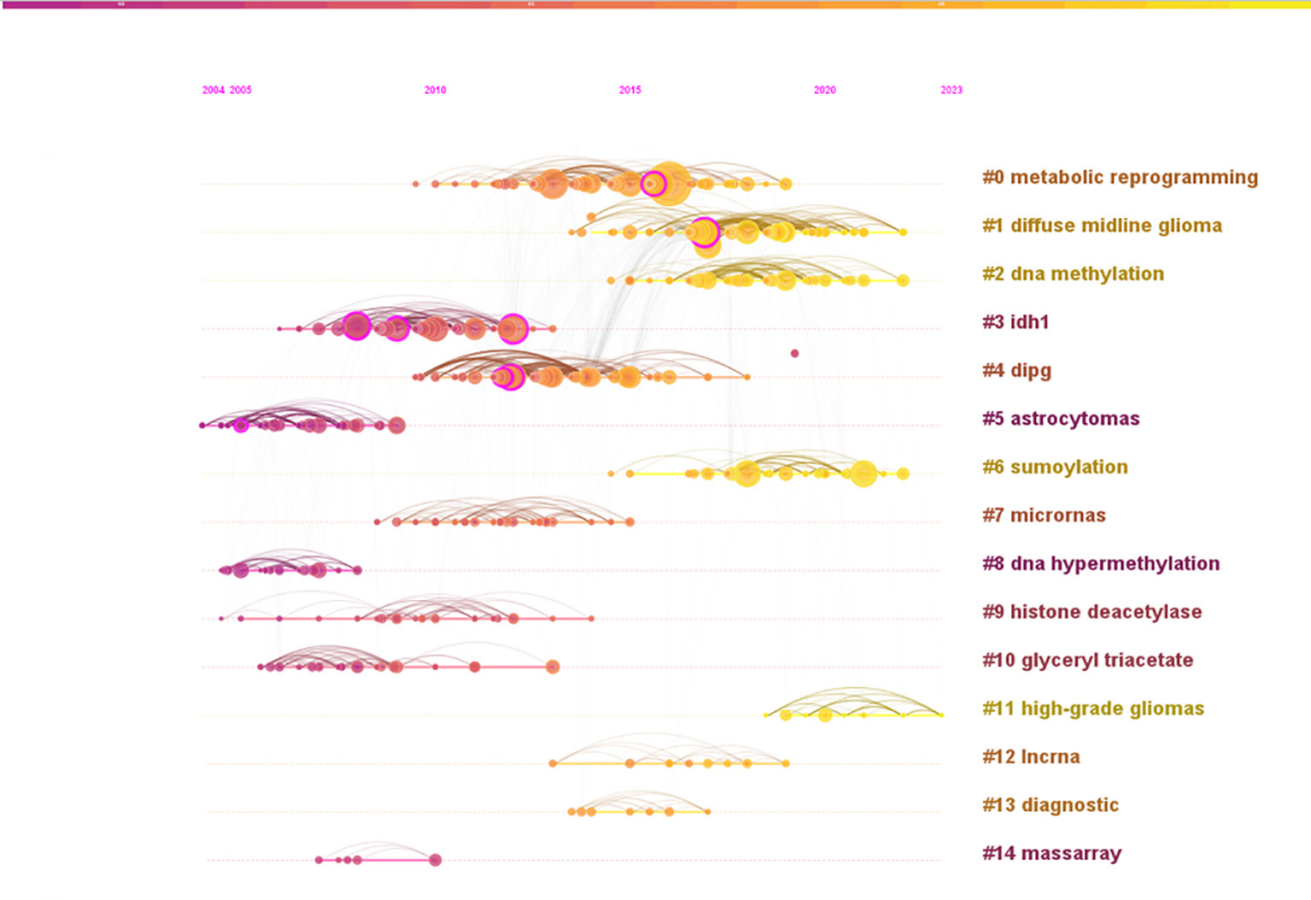
Analysis of references. A co-citation graph (timeline view) of references to publications related to epigenetic studies of gliomas.

Reference analysis plays a pivotal role in bibliometric studies. **Table 5** highlights the top 5 co-cited references based on centrality. The most central reference is the article titled “IDH1 and IDH2 mutations in gliomas”, authored by Yan H, and published in The New England Journal of Medicine in 2009 (24). This study identified mutations in the IDH1 gene and related IDH2 mutations in 445 central nervous system tumors and 494 non-central nervous system tumors. These findings suggest that mutations in NADP (+)-dependent isocitrate dehydrogenase, encoded by IDH1 and IDH2, could serve as a classification criterion for certain malignant gliomas. Further research indicated a potential link between one of the isocitrate dehydrogenase mutations and epigenetic inheritance (25). Another study published in “Cancer Research” titled “EMP3, a Myelin-Related Gene Located in the Critical 19q13.3 Region, is Epigenetically Silenced and Exhibits Features of a Candidate Tumor Suppressor in Glioma and Neuroblastoma” underscores the significance of EMP3 in epigenetic silencing and its potential role as a tumor suppressor in glioma and neuroblastoma. The co-citation analysis network identified 14 clusters, named by index terms as shown in **Figure 9**. The significance of the cluster structure is indicated by q values, where a q value greater than 0.3 is generally considered significant. In this study, the q value was 0.7449, and an s value greater than 0.7 is typically deemed convincing for clusters, with this study showing an s value of 0.9005. The largest cluster (#0) was “metabolic reprogramming”, followed by “DMG” (#1), “DNA methylation” (#2) and “IDH1” (#3).

**Table 5:** Top 5 most cited references from 2009 through January 2024.

Rank	Number	Centrality	Cited Reference	Years
1	27	0.18	Yan H, 2009, NEW ENGL J MED, V360, P765, DOI 10.1056/NEJMoa0808710	2009
2	17	0.16	Khuong-Quang DA, 2012, ACTA NEUROPATHOL, V124, P439, DOI 10.1007/s00401-012-0998-0	2012
3	5	0.13	Alaminos M, 2005, CANCER RES, V65, P2565, DOI 10.1158/0008-5472.CAN-04-4283	2005
4	36	0.13	Turcan S, 2012, NATURE, V483, P479, DOI 10.1038/nature10866	2012
5	31	0.12	Parsons DW, 2008, SCIENCE, V321, P1807, DOI 10.1126/science.1164382	2008

### 3.6 Keyword analysis

**Figure 10:**
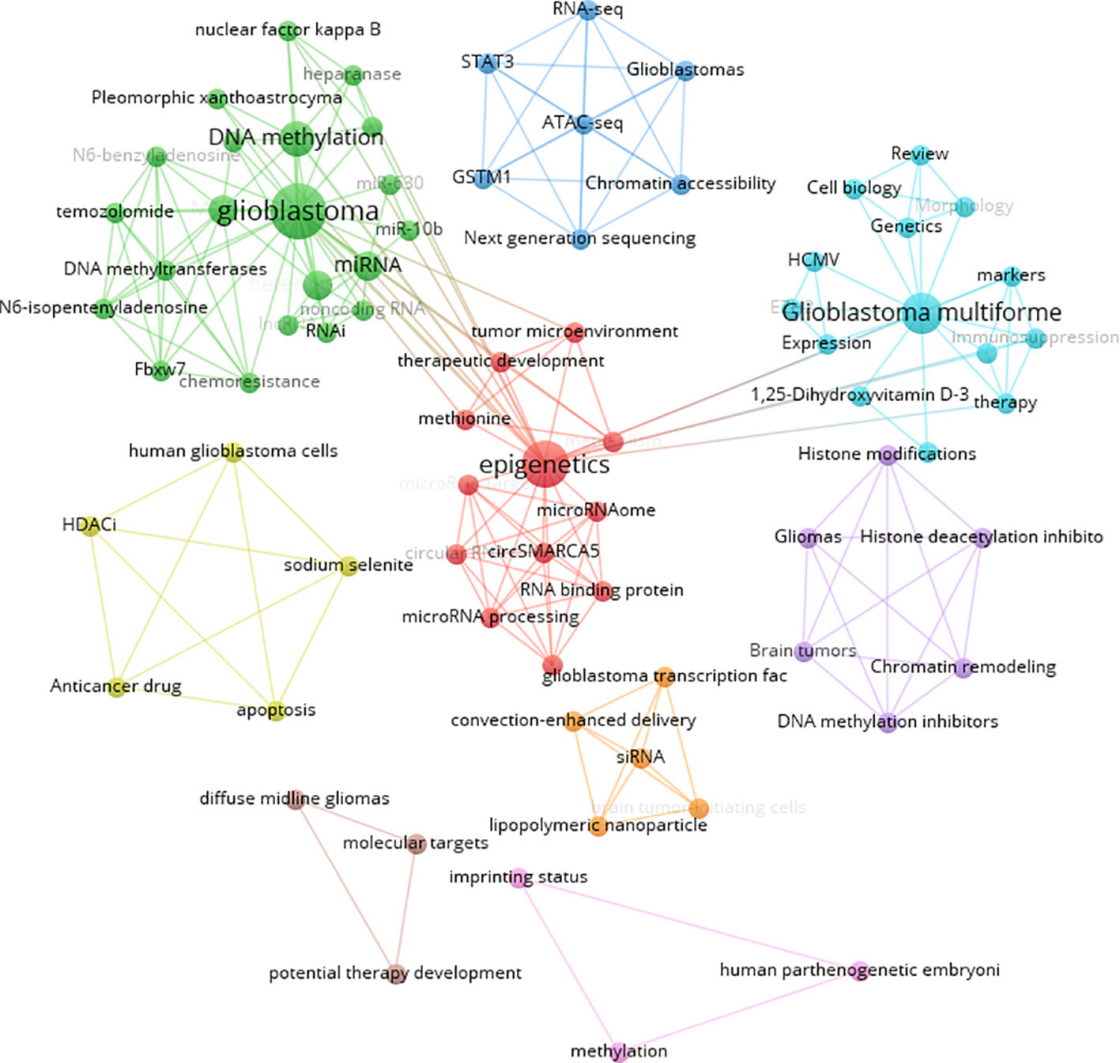
Visual mapping of the keywords.

**Figure 11:**
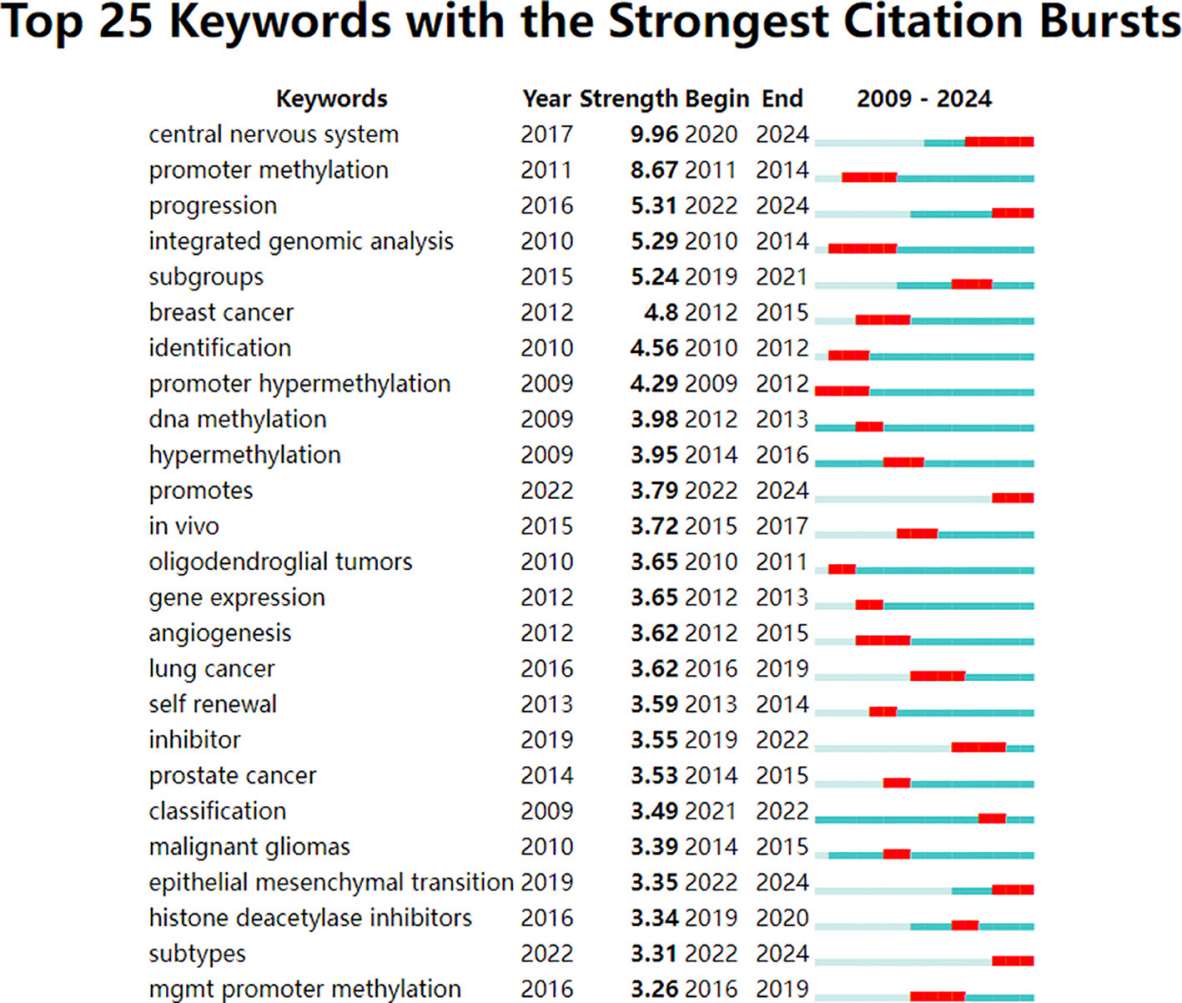
Top 25 keywords with the strongest citation bursts. The strongest citation bursts time period was indicated in red.

**Figure 12:**
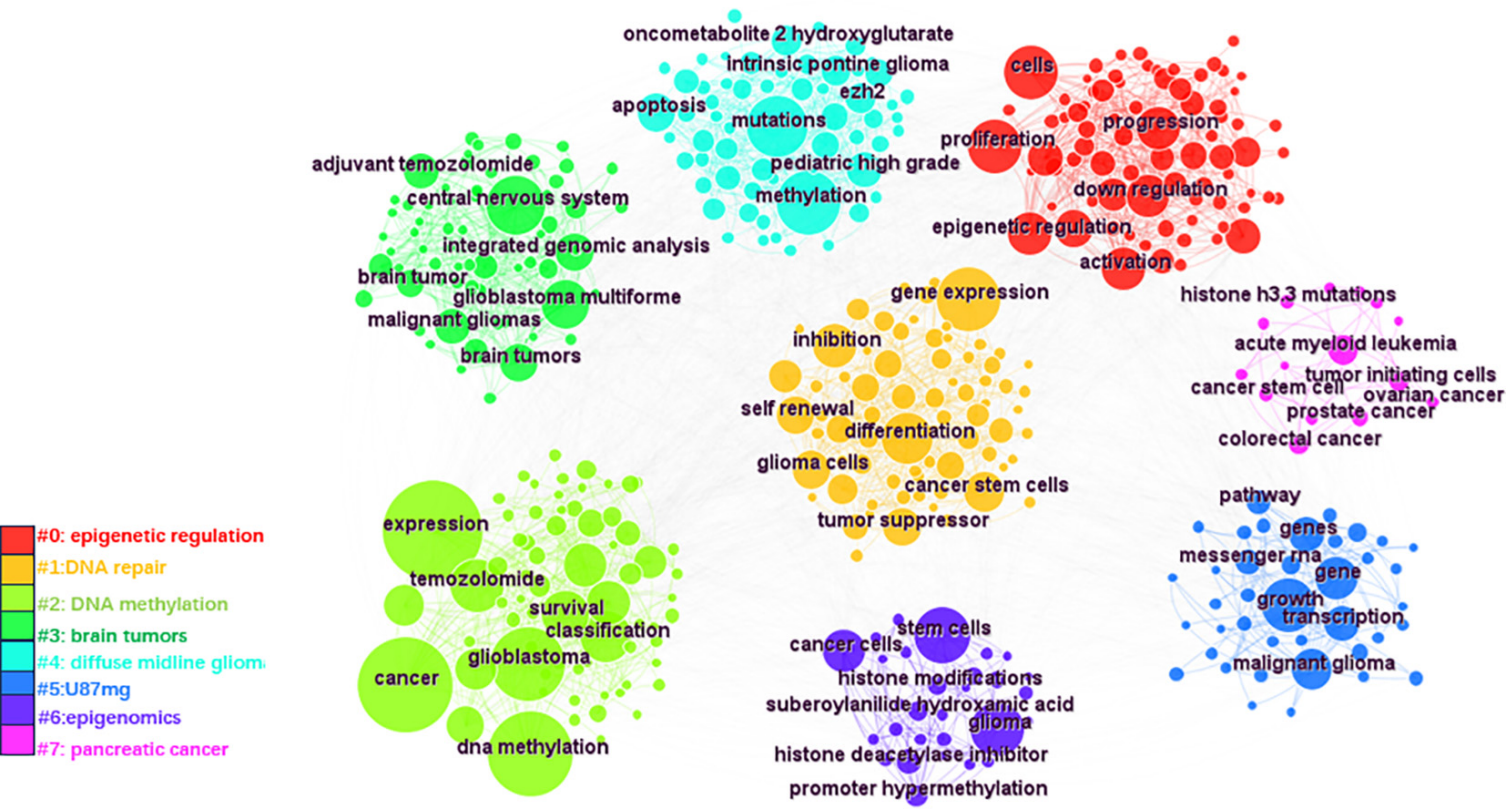
CiteSpace based keyword clustering. These keywords are divided into 7 categories by clustering and are shown in different color clusters. The topics of the thematic groups are shown in the corresponding legends.

Keywords were analyzed using CiteSpace to generate a keyword co-occurrence network diagram, as shown in **Figure 10**. After removing meaningless words, the ten most frequent keywords were “DNA methylation” (n=152), “GBM” (n=115), “mutations” (n=77), “stem cells” (n=64), “hypermethylation” (n=40), “proliferation” (n=59), “classification” (n=52), “differentiation” (n=71), “TMZ” (n=62), and “survival” (n=41). Notably, “DNA methylation”, “differentiation”, “GBM” and “stem cells” exhibited high centrality (**Table 6**). These top keywords can be categorized into two main groups: the first five focus on the epigenetic mechanisms of glioma, while the last five are related to glioma treatment. The analysis indicates that research on glioma epigenetics predominantly focuses on understanding the underlying mechanisms and exploring therapeutic options. **Figure 11** illustrates the top 25 keywords based on burst intensity, with “central nervous system” showing the highest burst intensity (9.96), followed by “promoter methylation” (8.67). Interestingly, besides keywords related to glioma epigenetics and treatment, the term “central nervous system” has also emerged as a popular topic. Furthermore, the study highlights specific types of gliomas and other common cancers, such as prostate, breast and lung cancers, indicating a wider range of interests in disease associations. Clustering analysis of co-occurring keywords revealed the main research themes in the field, as shown in **Figure 12**. The co-occurring keywords formed a total of seven clusters. Clusters #0 and #3 centered on potential epigenetic targets and specific therapies for gliomas. Key terms in these clusters included targeted therapy, protein kinase inhibitors, ion channels, proliferation, DNMT3a (DNA methyltransferase 3a), MIR-129-5P and TMZ. Cluster #1 focused on glioma genome-related content, including keywords such as EGFR/EGFRVIII, UBXN1, CRISPR/Cas9, NF-κB, differentiation and self-renewal. Cluster #2 addressed the prognosis and genetic factors of glioma, featuring topics like genetic abnormalities, epigenetic age, long-term survival, short-term survival and hypomethylation. Cluster #4 covered specific types of gliomas, such as diffuse midline glioma (DMG), pediatric diffuse glioma, hemispheric glioma and intrinsic pontine glioma. Clusters #5 and #6 explored potential mechanisms of epigenetic inheritance in gliomas, highlighting keywords like DNA methylation, chromatin modification, transcription, cancer cells, proliferation and migration. Lastly, Cluster #7 included discussions of other tumors.

**Table 6:** Top 10 keywords and their centrality.

Rank	Keywords	Counts	Centrality	Years
1	DNA methylation	152	0.05	2009
2	GBM	115	0.04	2012
3	Mutations	77	0.02	2009
4	Stem cells	64	0.03	2011
5	TMZ	62	0.02	2011
6	Proliferation	59	0.02	2014
7	Classification	52	0.01	2009
8	Differentiation	51	0.07	2011
9	Survival	47	0.03	2011
10	hypermethylation	40	0.04	2009

## 4 Discussion

### 4.1 General information

The distribution of time and annual volume of published literature provides valuable insights into the research hotspots and pace of development within the field of glioma epigenetics. By analyzing the publication volumes across four distinct time periods spanning from 2009 to 2024, we can gain a comprehensive understanding of the trends and shifts in this research area. Throughout these time periods, notable shifts in publication volumes were observed in 2016, 2019 and 2022. Despite these shifts, each period exhibited consistent stability in publication output, indicating a sustained level of research activity within the field. This stability, alongside an overall upward trend in publication volumes, underscores the increasing interest and importance of epigenetically related aspects of gliomas. The prominence of publications from specific countries, regions, or institutions further reflects their scientific contributions and capacity in this domain. The United States stands out as a leader in both the total number of publications and centrality, highlighting its prominent role in glioma epigenetics research. This dominance is indicative of the significant scientific resources and expertise dedicated to this field within the United States. Among the top authors in glioma epigenetics research, K Aldape holds the highest centrality, indicating a significant impact on the research community. Aldape’s contributions include a study identifying H3F3A and IDH1 hotspot mutations, which has defined an epigenetic subgroup of GBM. This research underscores the importance of understanding the epigenetic alterations in gliomas and their implications for disease classification and treatment. In conclusion, the analysis of the distribution of time and annual volume of published literature in glioma epigenetics research reveals consistent stability within each time period, alongside an overall upward trend. This trend underscores the increasing interest and importance of epigenetically related aspects of gliomas. The prominence of specific countries, regions, and institutions, as well as the significant contributions of key authors, further highlights the scientific capacity and advancements in this field (26). The epigenetic subgroup of GBM identified through the study of H3F3A and IDH1 hotspot mutations exhibits distinct global methylation patterns, DNA copy numbers, and transcriptome patterns. These unique features pave the way for the development of epigenetic-targeted therapies, offering new avenues for treating this aggressive brain tumor. Epigenetic markers, along with specific DNA alterations such as methyl-cytosine changes, low trimethylation of H3K27 (H3K27me3), reduced 5-methylcytosine (5mC), and elevated 5-hydroxymethylcytosine (5hmC), are emerging as key features of diffuse intrinsic pontine glioma (DIPG). This distinct epigenetic profile presents new opportunities for therapeutic interventions that target the underlying epigenetic alterations driving the disease. The imbalance between 5mC and 5hmC in DIPG is particularly noteworthy, as it suggests potential targets for therapeutic manipulation. By understanding the epigenetic mechanisms that contribute to the development and progression of DIPG, researchers can explore novel therapeutic strategies that specifically target these alterations, ultimately leading to improved patient outcomes. In conclusion, the identification of the epigenetic subgroup of GBM and the emerging features of DIPG provide new insights into the underlying mechanisms of these diseases. These distinct patterns and epigenetic markers offer promising opportunities for the development of targeted therapies that can address the specific alterations driving these gliomas. Further research in this area is crucial to advance our understanding of these complex diseases and to improve treatment options for patients (27). Keyword analysis is a critical method for unveiling research topics within the field. It illuminates trends such as epithelial-mesenchymal transition and the use of histone deacetylase inhibitors. IDH mutations are pivotal in the development of the CpG island methylator phenotype in gliomas, shedding light on mechanisms driving glioma development and intricate interplays between epigenetic modifications and genome-wide alterations (28). ncRNAs, which constitute crucial components of epigenetics, play a pivotal role in the development of glioma and the underlying mechanisms of resistance to TMZ (29). The co-citation analysis of references reveals the prevailing research focus in this field. Among the top 10 co-citations, the literature predominantly explores the discovery of specific therapeutic targets (30, 31), along with investigating the influence of related epigenetic factors on the diagnosis and detailed classification of gliomas (26, 28). As evidenced by analyzing authors, keywords and literature co-citations, both the investigation of epigenetic underpinnings in glioma genesis and the exploration of emerging therapeutic strategies represent pivotal focuses within the field of glioma epigenetics. Therefore, we will delve further into these two areas.

### 4.2 Epigenetic mechanisms

#### 4.2.1 DNA methylation

DNA methylation, a crucial epigenetic modification, has been extensively researched in the field of epigenetics. This process entails the addition of a methyl group to the 5th carbon atom of the cytosine ring, ultimately yielding 5mC (32). Specific methyltransferases recognize this modification, namely the addition of the methyl group to the cytosine ring, which subsequently plays a crucial role in regulating gene expression (33). In a study involving 20 glioma patients, Zhang et al. demonstrate that DNMT1-mediated methylation of Runx2 can impact miR-152, ultimately influencing glioma cell proliferation and apoptosis (34). Additionally, the reprogramming transcription factor Oct4 is found to promote GBM cell proliferation by directly activating the promoter of DNMT (35). DNA methylation plays a crucial role in regulating gene expression, and aberrant methylation patterns can lead to tumorigenesis by destabilizing tumor suppressor genes (36).

The dioxygenase family members, TET1, TET2, and TET3, can convert 5mC to 5hmC, which is the initial step in the process of DNA demethylation (37). IDH is an enzyme that is dependent on NAD+ and NADP+, and it plays a vital role in catalyzing the third step of the tricarboxylic acid cycle (38). Mutations in IDH1 have been linked to the accumulation of the metabolite 2-hydroxyglutarate (2-HG), which has the potential to induce the glioma-CpG island methylator phenotype (G-CIMP) by inhibiting TET-mediated production of 5hmC (19). The inhibition of TET activity represents a pivotal factor contributing to the observed abnormal DNA hypermethylation. This aberrant DNA methylation primarily affects promoter regions, where hypermethylation leads to the inactivation of tumor suppressor genes, whereas hypomethylation results in the activation of oncogenes (39). The O(6)-methylguanine-DNA methyltransferase (MGMT) gene, which is located on chromosome 10q26, is closely associated with DNA repair enzymes. Normal expression of MGMT significantly reduces the damage caused by alkylating chemotherapies in patients receiving such treatments (40). However, aberrant methylation of the MGMT gene promoter region in malignant gliomas leads to its inactivation. The activation of gene transcription necessitates the interaction of transcription factors with gene promoters, and the involvement of various transcription factors is crucial in the development of gliomas. Suva et al. have identified four transcription factors (SOX3, SALL2, OLIG2, and POU3F2) that are essential for the propagation of GBM (41). Preliminary experiments have demonstrated variations in mitochondrial DNA (mtDNA) copy numbers between glioma cells and healthy cells (42, 43). Subsequent studies suggest that epigenetic factors influence cell proliferation, apoptosis, and energy metabolism by regulating mtDNA gene expression, which is closely associated with the pathogenesis of gliomas (44). Further investigations have revealed that mtDNA methylation influences mtDNA transcriptional regulation and copy number variations. This alteration shifts the reliance of glioma cells from oxidative phosphorylation to glycolysis for ATP production, thereby promoting cell proliferation (45).

#### 4.2.2 Histone modifications

Histone modifications refer to alterations occurring at the amino terminus of histones during translation. These modifications encompass a wide range of processes such as phosphorylation, ubiquitination, acetylation, methylation and ADP-ribosylation (46). Recent research has underscored the pivotal role of histone modifications in the initiation and progression of human cancers (47). Histone acetylation, a process catalyzed by histone acetyltransferases (HATs), involves the covalent attachment of an acetyl group to specific lysine residues on histone proteins. This reaction occurs through the transfer of an acetyl group from acetyl-CoA to the hydrogen atoms of the ϵhemsge group of lysine, forming an N-acetyl-lysine linkage (48). The addition of acetyl groups to histone proteins disrupts the tight packaging of histone/DNA complexes within nucleosomes and subsequently impacts other interactions between nucleosomal histones (49). A study involving 70 human glioma samples revealed that histone deacetylation in the promoter regions of specific oncogenes, such as NECL1 and RRP22, led to reduced expression of these genes (50). Yu et al. demonstrated that hyperacetylation of histone H3 at lysine 9 (H3K9) contributed to abnormal hyper-transcription of the promoter region of the glial cell line-derived neurotrophic factor gene in glioma cells (51). Different levels of acetylation at specific sites on histone proteins can lead to diverse biological effects, which are indirectly influenced by changes in the associated enzymes that regulate histone acetylation levels and gene expression. Specifically, histone acetyltransferases (HATs) disrupt histone-DNA interactions, resulting in chromatin relaxation and facilitating access for transcription factors to the DNA (52). Conversely, histone deacetylases (HDACs) promote condensation of the chromatin, leading to transcriptional inhibition. Studies have found that HDAC inhibitors effectively prevent tumor progression by inducing cell death, cell cycle arrest, senescence, differentiation, and autophagy. For example, the HDAC inhibitor pracinostat has been shown to inhibit the acquisition of malignant features in brain tumors by upregulating TIMP3 expression and downregulating MMP2, MMP9 and VEGF in brain tumor cells (53, 54). In addition, HDAC inhibitors such as valproic acid and trichostatin A have been shown to enhance histone H4 acetylation and affect the biological behavior of glioma C6 cells (55).

Histone methylation involves the transfer of methyl groups from S-adenosylmethionine to histones, which is catalyzed by histone methyltransferases (HMTs) (56). The impact of histone methylation on transcription is influenced by various factors, including the type of histone, the specific modifying residues, and the extent of methylation at each site. For example, the methylation of H3K4, H3K36, and H3K79 is typically associated with active transcription in euchromatin, whereas the methylation of H3K9, H3K27 and H4K20 is linked to heterochromatic regions of the genome (57). Among the various histone modifications, H3K27me3 plays a crucial role in neural differentiation, particularly in the development of glioma. The PRC2 has been identified as a pivotal regulator of plasticity in glioma stem cell differentiation (58). The recruitment of PRC1 via the Chromobox (CBX) family proteins allows PRC2 to exhibit substrate specificity for H3K27, leading to the production of H3K27me3. Furthermore, H3K27 methylation promotes the binding of polycomb, a constituent of the PRC1 complex, to histone H3, thereby establishing a link between histone methylation and PcG-mediated gene silencing (59). The presence of H3K27M, in which methionine replaces the lysine residue at position 27, disrupts post-transcriptional silencing by inhibiting PRC2-mediated trimethylation, ultimately leading to increased histone hypomethylation (60). Conversely, the histone methyltransferase EZH2, a component of the PRC2 complex, catalyzes the methylation of histone H3 at lysine 27 (H3K27), ultimately resulting in the formation of H3K27me2 or H3K27me3. This epigenetic modification plays a crucial role in maintaining the progenitor state of neuroblastomas by silencing tumor suppressor genes, including CASZ1, CLU, RUNX3 and NGFR (61). Dysregulation of EZH2-H3K27me3 has been implicated in tumor progression, suggesting that EZH2-H3K27me3 represents a potential therapeutic target for gliomas (62, 63). The underlying mechanisms in the carcinogenesis led by alterations in EZH2 activity have been actively investigated (64). Moreover, up to 70% of secondary GBM patients harbor IDH1 mutations, which lead to neomorphic enzyme activity. This activity results in the production of 2-HG, an oncometabolite that inhibits αnhibitsbolite <EndNote>< dioxygenases, such as jumonji-C domain histone demethylases (JHDMs). This inhibition, due to the accumulation of 2-HG in malignant cells, promotes methylation (38, 65, 66). The accumulation of 2-HG plays a crucial role in the progression of LGGs to GBM, particularly in the presence of IDH1 mutations that are associated with H3K27- or H3K36-methylation (67). Therapeutic approaches targeting IDH mutations hold promise for enhancing glioma treatment, offering a potential new avenue for effective therapy (68).

#### 4.2.3 ncRNA dysregulation

A growing body of research has demonstrated that epigenetic alterations resulting from dysregulated ncRNAs play a significant role in influencing glioma progression (69). Long noncoding RNAs (lncRNAs), a distinct subtype of ncRNAs, play pivotal roles in a wide range of biological processes, notably including cancer progression (70). lncRNAs function as platforms that facilitate the localization of chromatin-modifying factors to specific genomic sites during gene expression. They can either redirect regulatory factors away from their intended targets or promote the spatial organization of chromosomes via epigenetic regulatory pathways. As an illustrative example, the lncRNA HOTAIRM1 has been shown to regulate HOXA1 gene expression by influencing key regulators, such as the demethylases G9a and EZH2, at transcriptional start sites. This regulation subsequently leads to a reduction in gene methylation and promotes the self-renewal and metastasis of glioma cells (71–73). Additionally, it has been reported that BRD4, a bromodomain-containing BET protein, binds to the promoter of the lncRNA HOTAIR (74). This interaction underscores the significance of structural domain proteins that target both the bromodomain and the extra terminal region as promising epigenetic regulators, offering new avenues for the treatment of gliomas (75). In addition to these elucidated mechanisms, lncRNAs can also influence disease progression through modulating protein activity, altering target interactions, and functioning as scaffolds for subcellular structures that bind to specific proteins, such as PCG, EZH and PRC22 (76).

**Figure 13:**
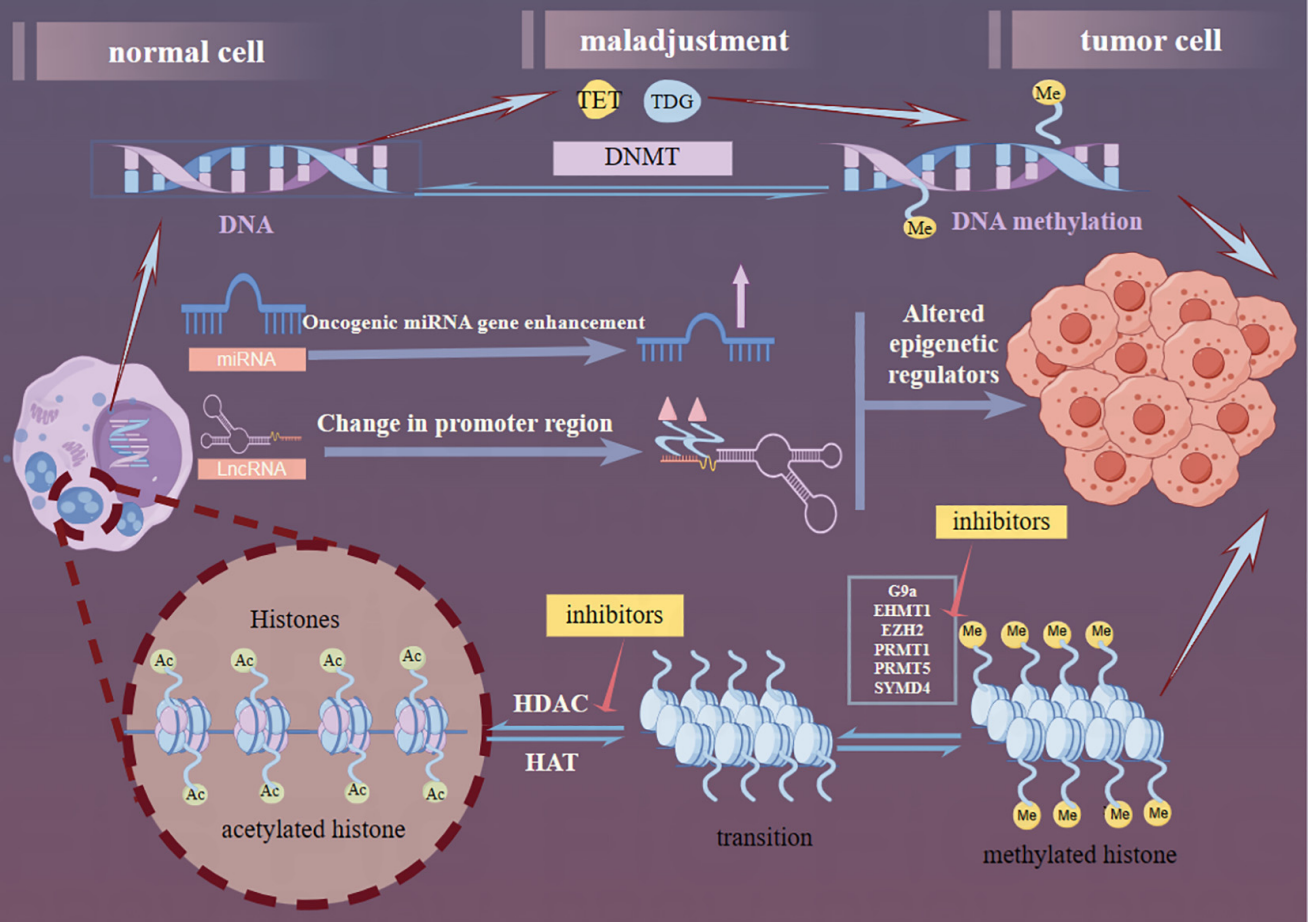
Graphical abstract. The epigenetic generation of gliomas consists of three components: DNA methylation, lncRNA dysregulation, and histone modifications. DNMT, the TET family, and the Thymine DNA glycosylase proteins are the current correlates of the factors that contribute to DNA methylation. In the ncRNA dysregulation section, miRNAs through enhancement of oncogenic miRNA genes and lncRNAs through alteration of promoter regions collectively affect gene regulation further altering the production of epigenetic regulators. In the histone modification part, reducing the activity of histone methyltransferase and HDAC to acetylate more sites in histone tails, thus reversing the aberrant histone modification, further inhibiting the proliferation and inducing apoptosis of tumor cells. Several targets have been used for glioma therapy (red arrows).

MicroRNAs (miRNAs), a class of endogenous ncRNAs with a length of 21-25 nucleotides, have recently been implicated in playing complex roles in glioma genesis. Specifically, the overexpression of oncogenic miRNAs, such as miR-128-3p or miR-145-5p, has been shown to induce anti-tumor effects in GBM (77). Conversely, the downregulation of oncogenic miRNAs, such as miR-21-3p or miR-21-5p, has been shown to prevent tumor progression in GBM. Furthermore, miRNAs also influence epitheliale,on.DATA ot transition (EMT) events by targeting ZEB regulators and other EMT-related factors. Specifically, in neural and glioma cells, miR-200c and miR-141 have been identified as inhibitors of cell growth and migration through their targeting of ZEB1 (78). Zinc finger E-box-binding homology box proteins, which play crucial roles in glioma developmental networks, represent potential therapeutic targets for intervention (79, 80). MiRNAs can be detected in the blood due to pathological changes in the BBB and have been proposed as biomarkers for central nervous system diseases (77). Overall, miRNAs play a pivotal role in glioma development, and continued research in this field holds great promise for the advancement of glioma treatment strategies. Recent studies have unveiled a reciprocal regulatory relationship between miRNAs and lncRNAs, where the decay of lncRNAs initiated by miRNAs influences the functional regulation of both miRNAs and lncRNAs (81). In gliomas, miRNAs and lncRNAs are primarily involved in the PI3K/Akt/mTOR, Wnt//Akt/mTOR, and Notch signaling pathways. Exploring the key molecules that connect these pathways offers a promising new direction for glioma research. **Figure 13** outlines these three key pathways of glioma epigenetics.

### 4.3 Research prospects and limitations

#### 4.3.1 Hot research topic

The frequency and intensity of keywords can provide insights into possible future developments in this field. As **Table 6** reveals, the keywords “GBM”, “TMZ” and “stem cells” rank highest in frequency, indicating that these topics dominate current research in the field of glioma epigenetics. Consequently, we will delve deeper into these three topics in our subsequent exploration.

##### 4.3.1.1 TMZ and GBM resistance

TMZ serves as the primary chemotherapeutic agent for treating GBM and has significantly enhanced patient survival rates. However, concerns regarding drug toxicity and resistance have become increasingly significant. Despite extensive research into resistance mechanisms and treatment strategies, clinical trials have yet to yield practical approaches for addressing TMZ resistance. Currently, the primary causes of TMZ resistance are believed to include the DNA repair system, autophagy, and glioma stem cells (GSCs). Specifically, three key DNA repair mechanisms are believed to contribute to TMZ resistance: (1) MGMT, (2) the mismatch repair (MMR) system, and (3) base excision repair (BER) via the poly (ADP-ribose) polymerase (PARP) pathway (82). If cells are deficient in MGMT, the primary resistance mechanism is directly related to high MGMT expression. In contrast, the secondary mechanism involves the mismatch repair (MMR) system in those cells (83–85). Targeting the PARP pathway involves the critical elimination of N7-methylguanine and N3-methyladenine adducts (86). TMZ-induced autophagy via the ATM/AMPK pathway can lead to the formation of autophagic vacuoles (AVOs) and the aggregation of LC3, both of which are essential for the interaction between autophagosomes and lysosomes. This interaction facilitates cytoprotective autophagy and promotes cell survival (87). GSCs can acquire resistance to TMZ by altering their phenotypes or differentiating into TMZ-resistant GBM cells through interactions with the tumor microenvironment, radiotherapy or hypoxic conditions (87). Furthermore, differentiated GBM cells may regain stem cell characteristics through modulation and reprogramming of the tumor microenvironment (88).

Besides the main TMZ resistance pathways mentioned previously, the significance of epigenetic factors in TMZ resistance is increasingly being recognized (89). Epigenetic alterations, specifically encompassing DNA methylation, histone modification, and chromatin remodeling, play pivotal roles in enhancing drug resistance and exacerbating mortality rates in GBM. These alterations contribute to therapeutic resistance through a multitude of mechanisms, which include promoting cell proliferation, inhibiting cell death, inducing stemness, impairing DNA damage repair mechanisms, and stimulating processes such as autophagy and EMT (90, 90). Recent research has demonstrated that the lncRNA SOX2OT plays a pivotal role in enhancing resistance to TMZ in GBM. This enhancement is achieved through the augmentation of SOX2 expression, which is mediated by ALKBH5-driven epigenetic mechanisms (91). Induced epigenetic modifications have been identified as key drivers of adaptive drug resistance in GBM (90). In addition to the aforementioned findings, it has been demonstrated that certain oncogenic lncRNAs, including H19, MALAT1, SNHGs, MIAT, UCA, HIF1A-AS2 and XIST, play a crucial role in regulating the invasion and metastasis of GBM cells. Specifically, lncRNA SBF2-AS1 has been identified as being closely associated with resistance to TMZ, highlighting its potential significance in the development of adaptive drug resistance in GBM (92). Consequently, ncRNAs, particularly lncRNAs, have been found to play critical roles in TMZ resistance, particularly from epigenetic perspectives. Exploring the mechanisms underlying the involvement of ncRNAs in TMZ resistance and their interactions with other epigenetic phenomena represents a promising direction for future research. Therefore, targeting epigenetic regulators in GBM patients holds potential clinical significance. At present, key epigenetic regulators that have been identified include histone methyltransferases (HMTs), histone demethylases, and histone deacetylases (90). A number of potential epigenetic-based therapeutic targets have been identified, including G9a, EHMT1, EZH2, PRMT1, PRMT5, and SYMD4 (17, 93). These epigenetic inhibitors have demonstrated not only the ability to target TMZ-resistant cells but also the capacity to directly inhibit the proliferation of GBM cells, highlighting their potential as therapeutic agents in GBM treatment (94–96). Furthermore, the combination of specific histone methyltransferase inhibitors with radiotherapy has shown potential to enhance tumor radiosensitivity, especially in tumors that exhibit significant treatment resistance, making this a promising approach for improving the efficacy of radiotherapy in GBM treatment (97). Therefore, continued research into epigenetic inhibitors may not only offer solutions for addressing TMZ resistance but also enhance drug efficacy for gliomas when combined with other therapeutic approaches, ultimately providing greater benefits to patients.

##### 4.3.1.2 Stem cells and GBM

GBM is the most prevalent and malignant primary brain tumor, with its high malignancy closely correlated with the presence of cancer stem cells (CSCs). These CSCs contribute to the occurrence and progression of GBM due to their strong tumorigenic, self-renewal, and multidirectional differentiation abilities (98). Epigenetic mechanisms are essential for maintaining normal stem and progenitor cells. Dysregulation of these mechanisms can result in the accumulation of cells with enhanced stemness properties and self-renewal capacity, potentially leading to the development of cancer stem cells CSCs (99). For example, GBM and other malignancies are composed of heterogeneous cancer cells, including glioblastoma-initiating cells (GICs). This heterogeneity in GBM and other malignancies is associated with various genetic mutations and epigenetic modifications that glioblastoma-initiating cells (GICs) acquire during their transformation (100).

Given the critical role of epigenetic mechanisms in regulating the properties of stem cells within cancer cells, targeting elements of these pathways could significantly contribute to eradicating CSCs and larger tumor populations. Currently, various therapeutic strategies are being proposed to address these mechanisms. Among them, inhibitors of epigenetic regulatory enzymes, such as HDACs and DNMTs, are being studied most extensively. Many of these inhibitors are currently undergoing clinical trials for the treatment of various cancers (99). Additionally, altered expression or activity of key epigenetic regulators can serve as prognostic indicators. For example, altered expression or activity of HDAC in GBM stem cells is associated with poor prognosis (101). Current experiments have shown that GSCs are particularly sensitive to the inhibition of histone demethylases KDM4C and KDM7A. This inhibition results in DNA damage and subsequent death of GSCs, while non-stem glioma cells remain unaffected (102). Currently, stem cell therapy is emerging as a viable alternative to the multimodal treatment of gliomas. In addition, stem cell therapy offers the advantages of tumor selectivity and targeted treatment, which can help to preserve healthy brain tissue (103). Therefore, ongoing epigenetic research focused on GSC transcription factors may unveil new therapeutic targets in the future.

#### 4.3.2 Emerging trends and new research fields

##### 4.3.2.1 Nano drug delivery systems with TMZ and epigenetics

Mechanisms of drug resistance pose a significant challenge in clinical practice, as they markedly limit the available treatment options for patients. Furthermore, the presence of the BBB further complicates therapeutic interventions, as it often reduces the effectiveness of drugs in reaching and treating conditions within the central nervous system, ultimately adversely affecting patient outcomes. These dual challenges underscore the need for continued research and development of novel strategies to overcome drug resistance and enhance drug delivery to the brain (104). Certain drugs, such as TMZ and paclitaxel, have the ability to cross the BBB, but they often require higher dosages due to their limited efficacy and permeability within the brain (105). Nano drug delivery systems (NDDS) could be the ideal solution to this problem (106). Currently, the use of target-responsive polymeric carriers has become a significant factor in selecting therapies for glioma, as they offer a more targeted and effective approach to delivering drugs to the brain (107). Furthermore, the limitations of immunotherapy, including local immune tolerance, the BBB, spatial heterogeneity, and the immune specificity of the glioma microenvironment, highlight the urgent need for more effective and targeted strategies to improve treatment outcomes (108). Combining immunotherapy with nanotechnology also emerges as a promising approach for glioma treatment. Current drug delivery methods encompass various types of carriers, including microspheres, biodegradable wafers, and colloidal drug carrier systems such as liposomes, exosomes and quantum dots, which offer improved targeting and delivery of therapeutic agents to the brain (109). It has been found that NDDS can enhance the targeting ability of TMZ. These systems can also control the release of TMZ, inhibit its degradation in the acidic environment of tumors and extend its biological half-life (110, 111). In recent years, experiments have been conducted to develop an effective NDDS for the chemotherapy of GBM using dopamine (PDA)-based, TMZ -loaded, Pep-1 (peptide-1)-coupled nanoparticles (NPs). This innovative approach aims to enhance the targeting and delivery of TMZ to GBM cells, ultimately improving treatment outcomes (112).

Among all NDDS, exosomes have garnered the most attention in recent decades. This is primarily due to their small size and the presence of natural protein and lipid components in the exosome membrane, which enable them to effectively penetrate biological barriers including the BBB and facilitate natural cellular uptake (113). Recent studies have revealed that exosomes can affect post-transcriptional regulation by involving in various epigenetic events. Additionally, certain ncRNAs, particularly miRNAs, have been shown to modulate cancer treatment resistance by regulating the expression of multiple oncogenes and tumor suppressors (114). Concurrently, exosomes hold promise as potential therapeutic and diagnostic tools, capable of carrying miRNAs and other compounds. These exosomes play a vital role in mediating intercellular communication during brain tumor development, thereby reflecting the progression of various brain pathologies (115–117). Currently, although therapeutic strategies for gliomas have achieved significant milestones, NDDS-based therapeutic strategies for gliomas are still in the preliminary clinical stage (118). For instance, certain drug delivery systems contend with endogenous substances within the body, which may subsequently lead to a reduction in their delivery efficiency (104). The development of multifunctional and multi-targeted NDDS is anticipated to be a prominent trend in the future of glioma nano-therapy. In the context of glioma nano-systems, especially from an epigenetic perspective, it is imperative to conduct further research on exosomes and comprehensively explore their role in epigenetic regulation.

##### 4.3.2.2 Photodynamic therapy with GBM and epigenetics

Over the past 50 years, the utilization of photosensitizers (PSs) in fluorescence-guided surgery and photodynamic therapy (PDT) for gliomas has experienced rapid expansion (119, 120). Compared to standard radiotherapy and chemotherapy, PDT selectively targets tumor tissue (121). The sensitivity and specificity for tumor targeting offered by PSs make PDT highly attractive for treating GBM. PDT exhibits high cytotoxicity against tumors while minimizing normal tissue toxicity and systemic effects, thereby reducing the risk of local recurrence (122, 123). Unlike TMZ, which exerts its therapeutic effect through DNA destabilization, photodynamic therapy (PDT) achieves tumor killing through oxidative damage to cell membranes, organelles, and proteins. This treatment modality induces a combination of apoptosis, necrosis, and autophagy (124).

In the realm of epigenetics, an experiment has used a straightforward and versatile strategy to change the subcellular localization of plasma membrane-targeted PDT PSs by amino acid modifications, aiming at precise tumor therapy (125). Meanwhile, histone deacetylase inhibitors have been discovered to enhance photodynamic therapy through the chromatin-based epigenetic regulation of CDKN1A in colon cancer cells (126). Is GBM photodynamic therapy also closely related to the discovery of a certain epigenetic regulatory mechanism? Furthermore, the combination of photodynamic therapy with epigenetic approaches has demonstrated potential in enhancing the efficacy of other treatment modalities. For instance, Ding and colleagues enhanced the efficacy of photoimmunotherapy by combining PDT with epigenetic therapy, which activates cellular pyroptosis and the cGAS-STING pathway in a light-controlled manner (127).

PDT presents a novel approach for managing malignant gliomas, offering potential solutions to various challenges in current treatments, particularly in targeted tumor therapy (128). However, the genetic and phenotypic diversity of gliomas suggests that single-agent PDT is unlikely to fully meet therapeutic needs, necessitating further studies. Moreover, research focusing on the epigenetic and photodynamic aspects of glioma treatment remains limited and warrants deeper exploration to fully understand its potential and limitations.

##### 4.3.2.3 Immunotherapy and glioblastoma epigenetics

Currently, ongoing research in GBM immunotherapy, which includes immune checkpoint blockade, chimeric antigen receptor T (CAR-T) cell therapy, oncolytic virus therapy (with more than 20 oncolytic virus candidates), and vaccine therapy, aims to minimize adverse side effects and enhance anti-tumor immune response through combination therapies (129, 130). Immunotherapy, as a systemic approach, has demonstrated promising potential in combating metastasis. However, it is acknowledged that current clinical immunotherapies are not universally effective across all patients or cancer metastasis types, primarily due to inadequate immune response. This underscores the need for further research to enhance immunotherapy efficacy and broaden its applicability in metastatic cancer treatment (131). This observation is particularly pertinent to GBM, a malignant tumor with a high risk of metastasis. Compared to other tumor types, GBM exhibits a relatively low number of somatic mutations and a notable lack of T-cell infiltration. These characteristics pose unique challenges for immunotherapy in GBM, highlighting the need for tailored strategies to enhance immune response and improve treatment outcomes (132). Indeed, the limited availability of immune checkpoint blockade in GBM, coupled with the fact that current immunotherapy approaches have not been successful, underscores the challenges faced in treating this malignancy. These limitations necessitate the exploration of novel immunotherapy strategies tailored to the unique features of GBM, with the aim of enhancing immune response and improving treatment outcomes (133). Despite the encouraging results from preclinical and phase I/II clinical trials, as well as success in some case reports, the transition to phase II/III remains particularly challenging in the context of GBM immunotherapy. This is evident by the fact that only a few vaccination approaches have been tested in phase III clinical trials for GBM patients, while many other immunotherapy approaches are still in the early stages of clinical development (129). To date, no successful phase III clinical trials of GBM immunotherapy targeting large patient cohorts have been reported (129), immunotherapy has a long way to go in the treatment of GBM (134).

Recent studies have elucidated epigenetic pathway regulation of GBM tumor expansion (93). By developing somatic mutations and epigenetic modifications, GBM tumor cells acquire the ability to counteract local immune responses (135). Meanwhile, the ability of glioma to evade immune surveillance and its resistance to therapy are attributed to epigenetic reprogramming of immune cells in the tumor microenvironment, which is induced by cancer metabolism. This reprogramming leads to an immunosuppressive state that facilitates tumor growth and progression, hindering the effectiveness of immunotherapy. Thus, elucidating the mechanisms underlying this epigenetic reprogramming and its interplay with cancer metabolism is essential for developing novel therapeutic strategies to overcome glioma’s immune evasion and resistance to treatment (136). In fact, a key factor in epigenetic regulation appears to be lncRNA that promote epigenetic regulatory molecular processes (137). lncRNA have been shown to control the activation and regulation of epigenetic enzymes (138), and participate in the resistance of cancer to immune response through antigen release, antigen presentation, immune activation, and immune cell migration and infiltration (139–141). Indeed, it is evident that epigenetic changes play a crucial role in the occurrence and development of GBM. Recent findings have shown that epigenetic regulation using GSK126, an EZH2 inhibitor, can improve current immunotherapy strategies by reversing the epigenetic changes that allow immune cells to evade, ultimately leading to enhanced transport of immune cells to tumors. This approach presents a promising new avenue for enhancing the effectiveness of immunotherapy in treating GBM and other cancers. By targeting epigenetic mechanisms, researchers hope to develop more effective and targeted therapies that can improve patient outcomes (142). Epigenetic modification factor JMJD6 has shown potential in modulating tumor immune response and may be an attractive target for novel tumor immunotherapy and prevention (143).

Therefore, further research in the field of glioma epigenetics is crucial for not only exploring the underlying causes of glioma occurrence and development but also identifying novel targets for immunotherapy. lncRNAs have emerged as key players in the field of epigenetics and warrant closer attention, not only in tumor treatment but also in prognosis. Studies have indeed shown that lncRNAs can serve as reliable prognostic and predictive tools to speculate which patients will benefit from adjuvant chemotherapy. By unraveling the roles of lncRNAs in glioma epigenetics, researchers can potentially uncover new avenues for targeted therapies and personalized treatment strategies, ultimately improving patient outcomes (141).

#### 4.3.3 Limitations

This pioneering bibliometric analysis provides valuable insights into the epigenetics of gliomas, offering an objective and quantitative evaluation of research trends and hotspots in the field. The findings have the potential to influence future research directions. However, the study has certain limitations. First, the data analyzed were solely from the WoSCC database, excluding other databases such as non-English databases, which may have led to the omission of relevant literature. Second, the filtering process using CiteSpace software was limited to SCI-Expanded from the WoSCC database. Finally, there is a possibility that some relevant literature lacking specific keywords may have been overlooked during the keyword-based search.

## 5 Conclusion

 This study conducted a bibliometric analysis of literature on glioma epigenetics from 2009 to 2024, revealing that GBM has been extensively studied among all glioma types in the realm of epigenetics. Research has particularly focused on mechanisms of TMZ resistance and overcoming therapeutic challenges, which remain current research hotspots. Continued in-depth exploration of epigenetic mechanisms such as DNA modifications, ncRNAs and histone modifications is crucial for identifying emerging targets. In the therapeutic arena, promising measures like nano-delivery systems, stem cell therapy, epigenetic immunology, and photodynamic therapy are emerging with momentum, facilitated by epigenetics. Furthermore, the exploration of epigenetic inhibitors and detailed study of exosomes represent future directions in glioma epigenetics research, holding potential for the development of novel therapeutic strategies and improved patient outcomes.

## Data availability statement

All the data generated or analyzed during this study are included in this paper. Further enquiries can be directed to the corresponding author.

## Glossary

WoSCC: Web of Science Core Collection
DMG: Diffuse Midline Glioma
ncRNAs: non-coding RNAs
TMZ: Temozolomide
WHO: World Health Organization
GBM: Glioblastoma
BBB: Blood‒Brain Barrier
ncRNAs: non-coding RNAs
IDH1: Isocitrate dehydrogenase 1
EMP3: Epithelial Membrane Protein 3
DNMT3a: DNA Methyltransferase 3a
EGFR/EGFRVIII: Epidermal Growth Factor Receptor or Epiderma Growth Factor Receptor 3
UBXN1: UBX Domain Protein 1
CRISPR/Cas9: Clustered Regularly Interspaced Short Palindromic Repeats or CRISPR-associated protein 9**;** nf-kb, *nuclear factor-k-gene binding*
H3F3A: *H3 histone, family 3A*
H3K27me3: Trimethylated Histone 3 at Lysine Residue 27
5mC: 5-methylcytosine
5hmC: 5-hydroxymethylcytosine
DIPG: Diffuse Intrinsic Pontine Glioma
DNMT1: DNA methyltransferase I
2-HG: 2-hydroxyglutarate
MGMT: O (6)-methylguanine-DNA methyltransferase
LGGs: Low-grade gliomas
GALNT9: Polypeptide N-acetylgalactosamine transferase 9
TMTC4: Transmembrane and tetratricopeptide repeat 4
SALL2: Spalt-like transcription factor 2
OLIG2: Oligodendrocyte transcription factor
POU3F2: POU-homeodomain transcription factor
CMYA5: Cardiomyopathy-Associated Protein 5
STEAP3: six-transmembrane epithelial antigen of prostat family member 3
mtDNA: mitochondrial DNA
ATP: adenosine triphosphate
NECL1: Nectin-like molecule 1
RRP22: Ras-related protein on chromosome 22
HATs: Histone acetyltransferases
HDACs: protein deacetylases
TIMP3: Tissue Inhibitor of Metalloproteinase 3
MMP2: Matrix Metalloproteinase-2
PRC2: Polycomb-repressive complex 2
H3K27: Histone 3, lysine 27
EZH2: Enhancer of zeste homolog 2
CASZ1: Castor zinc finger 1
CLU: Clusterin
RUNX3: Runt-related transcription factor 3
NGFR: Nerve growth factor receptor
JHDMs: Jumonji-C structural domain histone demethylases
HOTAIRM1: HOXA transcript antisense RNA myeloidspecific1
BRD4: Bromodomain Containing Protein 4
miRNAs: MicroRNAs
EMT: Epithelial–mesenchymal Transition
lncRNAs: Long noncoding RNAs
PI3K/Akt/mTOR: Phosphoinositide 3-kinase or AKT or mammalian target of rapamycin
ATM/AMPK: Ataxia-telangiectasia mutated or PurposeAMP-Activated protein kinase
HMTs: histone methyltransferases
MALAT1: Metastasis-associated lung adenocarcinoma transcript 1
SNHGs: Small nucleolar RNA host genes
MIAT: Myocardial infarction-associated transcript
HIF1A-AS2: HIF1A antisense RNA 2
XIST: X inactive-specific transcript
EHMT1: Euchromatic histone-lysine N-methyltransferase 1
NDDS: nano drug delivery systems
CSC: Cancer Stem Cells
GICs: GBM-initiating cells
KDM4C: Lysine demethylase 4C
KDM7A: Lysine demethylase 7A
PSs: Photosensitisers
PDT: Photodynamic therapy
CDKN1A: Cyclin-dependent kinase inhibitor 1A

## References

[1] Weller M Wick W Aldape K Brada M Berger M Pfister SM . Glioma. Nat Rev Dis Primers. (2015) 1:15017. doi: 10.1038/nrdp.2015.17 27188790

[2] IARC . Home . Available online at: https://www.iarc.who.int/.

[3] Teraiya M Perreault H Chen VC . An overview of glioblastoma multiforme and temozolomide resistance: can LC-MS-based proteomics reveal the fundamental mechanism of temozolomide resistance? Front Oncol. (2023) 13:1166207. doi: 10.3389/fonc.2023.1166207 37182181 PMC10169742

[4] Agnez-Lima LF Melo JT Silva AE Oliveira AH Timoteo AR Lima-Bessa KM . DNA damage by singlet oxygen and cellular protective mechanisms. Mutat Res Rev Mutat Res. (2012) 751:15–28. doi: 10.1016/j.mrrev.2011.12.005 22266568

[5] Ostrom QT Cioffi G Waite K Kruchko C Barnholtz-Sloan JS . CBTRUS statistical report: primary brain and other central nervous system tumors diagnosed in the United States in 2014-2018. Neuro Oncol. (2021) 23:iii1–iii105. doi: 10.1093/neuonc/noab200 34608945 PMC8491279

[6] Zang L Kondengaden SM Che F Wang L Heng X . Potential epigenetic-based therapeutic targets for glioma. Front Mol Neurosci. (2018) 11:408. doi: 10.3389/fnmol.2018.00408 30498431 PMC6249994

[7] Louis DN Perry A Wesseling P Brat DJ Cree IA Figarella-Branger D . The 2021 WHO classification of tumors of the central nervous system: a summary. Neuro Oncol. (2021) 23:1231–51. doi: 10.1093/neuonc/noab106 PMC8328013 34185076

[8] Low JT Ostrom QT Cioffi G Neff C Waite KA Kruchko C . Primary brain and other central nervous system tumors in the United States (2014-2018): A summary of the CBTRUS statistical report for clinicians. Neurooncol Pract. (2022) 9:165–82. doi: 10.1093/nop/npac015 PMC9113389 35601966

[9] Stupp R Hegi ME Mason WP van den Bent MJ Taphoorn MJ Janzer RC . Effects of radiotherapy with concomitant and adjuvant temozolomide versus radiotherapy alone on survival in glioblastoma in a randomised phase III study: 5-year analysis of the EORTC-NCIC trial. Lancet Oncol. (2009) 10:459–66. doi: 10.1016/S1470-2045(09)70025-7 19269895

[10] Stupp R Mason WP van den Bent MJ Weller M Fisher B Taphoorn MJ . Radiotherapy plus concomitant and adjuvant temozolomide for glioblastoma. N Engl J Med. (2005) 352:987–96. doi: 10.1056/NEJMoa043330 15758009

[11] Majewska P Ioannidis S Raza MH Tanna N Bulbeck H Williams M . Postprogression survival in patients with glioblastoma treated with concurrent chemoradiotherapy: a routine care cohort study. CNS Oncol. (2017) 6:307–13. doi: 10.2217/cns-2017-0001 PMC6004888 28990795

[12] Nabors LB Portnow J Ammirati M Baehring J Brem H Butowski N . NCCN guidelines insights: central nervous system cancers, version 1.2017. J Natl Compr Canc Netw. (2017) 15:1331–45. doi: 10.6004/jnccn.2017.0166 29118226

[13] Hadjipanayis CG Van Meir EG . Tumor initiating cells in Malignant gliomas: biology and implications for therapy. J Mol Med (Berl). (2009) 87:363–74. doi: 10.1007/s00109-009-0440-9 PMC2693383 19189072

[14] Lee SY . Temozolomide resistance in glioblastoma multiforme. Genes Dis. (2016) 3:198–210. doi: 10.1016/j.gendis.2016.04.007 30258889 PMC6150109

[15] Fath MK Babakhaniyan K Anjomrooz M Jalalifar M Alizadeh SD Pourghasem Z . Recent advances in glioma cancer treatment: conventional and epigenetic realms. Vaccines. (2022) 10:1448. doi: 10.3390/vaccines10091448 36146527 PMC9501259

[16] Nicholson JG Fine HA . Diffuse glioma heterogeneity and its therapeutic implications. Cancer Discovery. (2021) 11:575–90. doi: 10.1158/2159-8290.CD-20-1474 33558264

[17] Gusyatiner O Hegi ME . Glioma epigenetics: From subclassification to novel treatment options. Semin Cancer Biol. (2018) 51:50–8. doi: 10.1016/j.semcancer.2017.11.010 29170066

[18] Dawson MA Kouzarides T . Cancer epigenetics: from mechanism to therapy. Cell. (2012) 150:12–27. doi: 10.1016/j.cell.2012.06.013 22770212

[19] Kondo Y Katsushima K Ohka F Natsume A Shinjo K . Epigenetic dysregulation in glioma. Cancer Sci. (2014) 105:363–9. doi: 10.1111/cas.2014.105.issue-4 PMC4317798 24843883

[20] Pop S Enciu AM Necula LG Tanase C . Long non-coding RNAs in brain tumours: Focus on recent epigenetic findings in glioma. J Cell Mol Med. (2018) 22:4597–610. doi: 10.1111/jcmm.2018.22.issue-10 PMC6156469 30117678

[21] Merigó JM Gil-Lafuente AM Yager RR . An overview of fuzzy research with bibliometric indicators. Appl Soft Computing. (2015) 27:420–33. doi: 10.1016/j.asoc.2014.10.035

[22] Chen C . Searching for intellectual turning points: progressive knowledge domain visualization. Proc Natl Acad Sci U S A. (2004) 101 Suppl 1:5303–10. doi: 10.1073/pnas.0307513100 PMC387312 14724295

[23] Bu Y Liu TY Huang WB . MACA: a modified author co-citation analysis method combined with general descriptive metadata of citations. Scientometrics. (2016) 108:143–66. doi: 10.1007/s11192-016-1959-5

[24] Yan H Parsons DW Jin G McLendon R Rasheed BA Yuan W . IDH1 and IDH2 mutations in gliomas. N Engl J Med. (2009) 360:765–73. doi: 10.1056/NEJMoa0808710 PMC2820383 19228619

[25] Braun Y Filipski K Bernatz S Baumgarten P Roller B Zinke J . Linking epigenetic signature and metabolic phenotype in IDH mutant and IDH wildtype diffuse glioma. Neuropathol Appl Neurobiol. (2021) 47:379–93. doi: 10.1111/nan.12669 33080075

[26] Sturm D Witt H Hovestadt V Khuong-Quang DA Jones DTW Konermann C . Hotspot mutations in H3F3A and IDH1 define distinct epigenetic and biological subgroups of glioblastoma. Cancer Cell. (2012) 22:425–37. doi: 10.1016/j.ccr.2012.08.024 23079654

[27] Ahsan S Raabe EH Haffner MC Vaghasia A Warren KE Quezado M . Increased 5-hydroxymethylcytosine and decreased 5-methylcytosine are indicators of global epigenetic dysregulation in diffuse intrinsic pontine glioma. Acta Neuropathol Commun. (2014) 2:59. doi: 10.1186/2051-5960-2-59 24894482 PMC4229804

[28] Turcan S Rohle D Goenka A Walsh LA Fang F Yilmaz E . IDH1 mutation is sufficient to establish the glioma hypermethylator phenotype. Nature. (2012) 483:479–U137. doi: 10.1038/nature10866 22343889 PMC3351699

[29] Rezaee A Tehrany PM Tirabadi FJ Sanadgol N Karimi AS Ajdari A . Epigenetic regulation of temozolomide resistance in human cancers with an emphasis on brain tumors: Function of non-coding RNAs. BioMed Pharmacother. (2023) 165:115187. doi: 10.1016/j.biopha.2023.115187 37499452

[30] Mohammad F Weissmann S Leblanc B Pandey DP Højfeldt JW Comet I . EZH2 is a potential therapeutic target for H3K27M-mutant pediatric gliomas. Nat Med. (2017) 23:483–92. doi: 10.1038/nm.4293 28263309

[31] Piunti A Hashizume R Morgan MA Bartom ET Horbinski CM Marshall SA . Therapeutic targeting of polycomb and BET bromodomain proteins in diffuse intrinsic pontine gliomas. Nat Med. (2017) 23:493–500. doi: 10.1038/nm.4296 28263307 PMC5667640

[32] Angeloni A Bogdanovic O . Enhancer DNA methylation: implications for gene regulation. Essays Biochem. (2019) 63:707–15. doi: 10.1042/EBC20190030 31551326

[33] Lv H Dao FY Zhang D Yang H Lin H . Advances in mapping the epigenetic modifications of 5-methylcytosine (5mC), N6-methyladenine (6mA), and N4-methylcytosine (4mC). Biotechnol Bioeng. (2021) 118:4204–16. doi: 10.1002/bit.v118.11 34370308

[34] Zhang P Sun H Yang B Luo W Liu Z Wang J . miR-152 regulated glioma cell proliferation and apoptosis via Runx2 mediated by DNMT1. BioMed Pharmacother. (2017) 92:690–5. doi: 10.1016/j.biopha.2017.05.096 28595085

[35] Lopez-Bertoni H Lal B Li A Caplan M Guerrero-Cázares H Eberhart CG . DNMT-dependent suppression of microRNA regulates the induction of GBM tumor-propagating phenotype by Oct4 and Sox2. Oncogene. (2015) 34:3994–4004. doi: 10.1038/onc.2014.334 25328136 PMC4404208

[36] Meng H Cao Y Qin J Song X Zhang Q Shi Y . DNA methylation, its mediators and genome integrity. Int J Biol Sci. (2015) 11:604–17. doi: 10.7150/ijbs.11218 PMC4400391 25892967

[37] Joshi K Liu S Breslin SJP Zhang J . Mechanisms that regulate the activities of TET proteins. Cell Mol Life Sci. (2022) 79:363. doi: 10.1007/s00018-022-04396-x 35705880 PMC9756640

[38] Figueroa ME Abdel-Wahab O Lu C Ward PS Patel J Shih A . Leukemic IDH1 and IDH2 mutations result in a hypermethylation phenotype, disrupt TET2 function, and impair hematopoietic differentiation. Cancer Cell. (2010) 18:553–67. doi: 10.1016/j.ccr.2010.11.015 PMC4105845 21130701

[39] Biserova K Jakovlevs A Uljanovs R Strumfa I . Cancer stem cells: significance in origin, pathogenesis and treatment of glioblastoma. Cells. (2021) 10:621. doi: 10.3390/cells10030621 33799798 PMC8000844

[40] Riemenschneider MJ Hegi ME Reifenberger G . *MGMT* promoter methylation in Malignant gliomas. Targeted Oncol. (2010) 5:161–5. doi: 10.1007/s11523-010-0153-6 20725792

[41] Suvà ML Rheinbay E Gillespie SM Patel AP Wakimoto H Rabkin SD . Reconstructing and reprogramming the tumor-propagating potential of glioblastoma stem-like cells. Cell. (2014) 157:580–94. doi: 10.1016/j.cell.2014.02.030 PMC4004670 24726434

[42] Liang BC Hays L . Mitochondrial DNA copy number changes in human gliomas. Cancer Lett. (1996) 105:167–73. doi: 10.1016/0304-3835(96)04276-0 8697440

[43] Lai RK Chen Y Guan X Nousome D Sharma C Canoll P . Genome-wide methylation analyses in glioblastoma multiforme. PloS One. (2014) 9:e89376. doi: 10.1371/journal.pone.0089376 24586730 PMC3931727

[44] Grady CI Walsh LM Heiss JD . Mitoepigenetics and gliomas: epigenetic alterations to mitochondrial DNA and nuclear DNA alter mtDNA expression and contribute to glioma pathogenicity. Front Neurol. (2023) 14. doi: 10.3389/fneur.2023.1154753 PMC10270738 37332990

[45] Lee W Johnson J Gough DJ Donoghue J Cagnone GL Vaghjiani V . Mitochondrial DNA copy number is regulated by DNA methylation and demethylation of POLGA in stem and cancer cells and their differentiated progeny. Cell Death Dis. (2015) 6:e1664. doi: 10.1038/cddis.2015.34 25719248 PMC4669800

[46] Lennartsson A Ekwall K . Histone modification patterns and epigenetic codes. Biochim Et Biophys Acta-General Subjects. (2009) 1790:863–8. doi: 10.1016/j.bbagen.2008.12.006 19168116

[47] Wang RL Xin M Li YJ Zhang PY Zhang MX . The functions of histone modification enzymes in cancer. Curr Protein Pept Sci. (2016) 17:438–45. doi: 10.2174/1389203717666160122120521 26796305

[48] Tao HH Li QH Lin YX Zuo HY Cui Y Chen S . Coordinated expression of p300 and HDAC3 upregulates histone acetylation during dentinogenesis. J Cell Biochem. (2020) 121:2478–88. doi: 10.1002/jcb.v121.3 PMC7808212 31692090

[49] Koprinarova M Schnekenburger M Diederich M . Role of histone acetylation in cell cycle regulation. Curr Topics Med Chem. (2016) 16:732–44. doi: 10.2174/1568026615666150825140822 26303420

[50] Schmidt N Windmann S Reifenberger G Riemenschneider MJ . DNA hypermethylation and histone modifications downregulate the candidate tumor suppressor gene RRP22 on 22q12 in human gliomas. Brain Pathol. (2012) 22:17–25. doi: 10.1111/j.1750-3639.2011.00507.x 21631628 PMC8028980

[51] Yu Z Zhang B Ni H Liu Z Wang J Ren Q . Hyperacetylation of histone H3K9 involved in the promotion of abnormally high transcription of the gdnf gene in glioma cells. Mol Neurobiol. (2014) 50:914–22. doi: 10.1007/s12035-014-8666-0 24619502

[52] Grayson DR Kundakovic M Sharma RP . Is there a future for histone deacetylase inhibitors in the pharmacotherapy of psychiatric disorders? Mol Pharmacol. (2010) 77:126–35. doi: 10.1124/mol.109.061333 19917878

[53] Falkenberg KJ Johnstone RW . Histone deacetylases and their inhibitors in cancer, neurological diseases and immune disorders. Nat Rev Drug Discovery. (2014) 13:673–91. doi: 10.1038/nrd4360 25131830

[54] Chen M Zhang L Zhan R Zheng X . The novel histone deacetylase inhibitor pracinostat suppresses the Malignant phenotype in human glioma. Mol Biol Rep. (2022) 49:7507–19. doi: 10.1007/s11033-022-07559-y 35622308

[55] Maleszewska M Steranka A Kaminska B . The effects of selected inhibitors of histone modifying enzyme on C6 glioma cells. Pharmacol Rep. (2014) 66:107–13. doi: 10.1016/j.pharep.2013.08.011 24905315

[56] Niu Y Bai J Zheng S . The regulation and function of histone methylation. J Plant Biol. (2018) 61:347–57. doi: 10.1007/s12374-018-0176-6

[57] Barski A Cuddapah S Cui K Roh TY Schones DE Wang Z . High-resolution profiling of histone methylations in the human genome. Cell. (2007) 129:823–37. doi: 10.1016/j.cell.2007.05.009 17512414

[58] Natsume A Ito M Katsushima K Ohka F Hatanaka A Shinjo K . Chromatin regulator PRC2 is a key regulator of epigenetic plasticity in glioblastoma. Cancer Res. (2013) 73:4559–70. doi: 10.1158/0008-5472.CAN-13-0109 23720055

[59] Cao R Wang L Wang H Xia L Erdjument-Bromage H Tempst P . Role of histone H3 lysine 27 methylation in Polycomb-group silencing. Science. (2002) 298:1039–43. doi: 10.1126/science.1076997 12351676

[60] Cooney TM Lubanszky E Prasad R Hawkins C Mueller S . Diffuse midline glioma: review of epigenetics. J Neurooncol. (2020) 150:27–34. doi: 10.1007/s11060-020-03553-1 32804378

[61] Wang C Liu Z Woo CW Li Z Wang L Wei JS . EZH2 Mediates epigenetic silencing of neuroblastoma suppressor genes CASZ1, CLU, RUNX3, and NGFR. Cancer Res. (2012) 72:315–24. doi: 10.1158/0008-5472.CAN-11-0961 PMC3487161 22068036

[62] Ohka F Natsume A Suzuki H Aoki K Deguchi S Katsushima K . Targeting dysregulation of EZH2-H3K27me3 as an effective treatment for IDH-wildtype lower grade glioma. Cancer Sci. (2018) 109:463–.

[63] Ohka F Deguchi S Suzuki H Aoki K Katsushima K Shinjo K . Epigenomic treatment for IDH wild-type grade III glioma, targeting dysregulation of ezh2-h3k27me3. Neuro-Oncology. (2016) 18:66–. doi: 10.1093/neuonc/now212.278

[64] Margueron R Reinberg D . The Polycomb complex PRC2 and its mark in life. Nature. (2011) 469:343–9. doi: 10.1038/nature09784 PMC3760771 21248841

[65] Xu W Yang H Liu Y Yang Y Wang P Kim S-H . Oncometabolite 2-hydroxyglutarate is a competitive inhibitor of αfhibitorveutarate-9.3./dioxygenases. Cancer Cell. (2011) 19:17–30. doi: 10.1016/j.ccr.2010.12.014 21251613 PMC3229304

[66] McBrayer SK Mayers JR DiNatale GJ Shi DD Khanal J Chakraborty AA . Transaminase inhibition by 2-hydroxyglutarate impairs glutamate biosynthesis and redox homeostasis in glioma. Cell. (2018) 175:101–16.e25. doi: 10.1016/j.cell.2018.08.038 30220459 PMC6219629

[67] Williams MJ Singleton WG Lowis SP Malik K Kurian KM . Therapeutic targeting of histone modifications in adult and pediatric high-grade glioma. Front Oncol. (2017) 7:45. doi: 10.3389/fonc.2017.00045 28401060 PMC5368219

[68] Persico P Lorenzi E Losurdo A Dipasquale A Di Muzio A Navarria P . Precision oncology in lower-grade gliomas: promises and pitfalls of therapeutic strategies targeting IDH-mutations. Cancers (Basel). (2022) 14:1125. doi: 10.3390/cancers14051125 35267433 PMC8909346

[69] Rynkeviciene R Simiene J Strainiene E Stankevicius V Usinskiene J Kaubriene EM . Non-coding RNAs in glioma. Cancers. (2019) 11:219. doi: 10.3389/fgene.2012.00219 30583549 PMC6356972

[70] Tano K Akimitsu N . Long non-coding RNAs in cancer progression. Front Genet. (2012) 3:219. doi: 10.3389/fgene.2012.00219 23109937 PMC3479403

[71] Jain AK Xi Y McCarthy R Allton K Akdemir KC Patel LR . LncPRESS1 is a p53-regulated lncRNA that safeguards pluripotency by disrupting SIRT6-mediated de-acetylation of histone H3K56. Mol Cell. (2016) 64:967–81. doi: 10.1016/j.molcel.2016.10.039 PMC5137794 27912097

[72] Li W Notani D Ma Q Tanasa B Nunez E Chen AY . Functional roles of enhancer RNAs for oestrogen-dependent transcriptional activation. Nature. (2013) 498:516–20. doi: 10.1038/nature12210 PMC3718886 23728302

[73] Xia H Liu Y Wang Z Zhang W Qi M Qi B . Long noncoding RNA HOTAIRM1 maintains tumorigenicity of glioblastoma stem-like cells through regulation of HOX gene expression. Neurotherapeutics. (2020) 17:754–64. doi: 10.1007/s13311-019-00799-0 PMC7283434 31691127

[74] Pastori C Kapranov P Penas C Peschansky V Volmar CH Sarkaria JN . The Bromodomain protein BRD4 controls HOTAIR, a long noncoding RNA essential for glioblastoma proliferation. Proc Natl Acad Sci U S A. (2015) 112:8326–31. doi: 10.1073/pnas.1424220112 PMC4500283 26111795

[75] Filippakopoulos P Knapp S . Targeting bromodomains: epigenetic readers of lysine acetylation. Nat Rev Drug Discovery. (2014) 13:337–56. doi: 10.1038/nrd4286 24751816

[76] Wilusz JE Sunwoo H Spector DL . Long noncoding RNAs: functional surprises from the RNA world. Genes Dev. (2009) 23:1494–504. doi: 10.1101/gad.1800909 PMC3152381 19571179

[77] Petrescu GED Sabo AA Torsin LI Calin GA Dragomir MP . MicroRNA based theranostics for brain cancer: basic principles. J Exp Clin Cancer Res. (2019) 38:231. doi: 10.1186/s13046-019-1180-5 31142339 PMC6542029

[78] Guo E Wang Z Wang S . MiR-200c and miR-141 inhibit ZEB1 synergistically and suppress glioma cell growth and migration. Eur Rev Med Pharmacol Sci. (2016) 20:3385–91. 27608897

[79] Depner C Zum Buttel H Böğürcü N Cuesta AM Aburto MR Seidel S . EphrinB2 repression through ZEB2 mediates tumour invasion and anti-angiogenic resistance. Nat Commun. (2016) 7:12329. doi: 10.1038/ncomms12329 27470974 PMC4974575

[80] Qi S Song Y Peng Y Wang H Long H Yu X . ZEB2 mediates multiple pathways regulating cell proliferation, migration, invasion, and apoptosis in glioma. PloS One. (2012) 7:e38842. doi: 10.1371/journal.pone.0038842 22761708 PMC3383704

[81] Yoon JH Abdelmohsen K Gorospe M . Functional interactions among microRNAs and long noncoding RNAs. Semin Cell Dev Biol. (2014) 34:9–14. doi: 10.1016/j.semcdb.2014.05.015 24965208 PMC4163095

[82] Zhang J Stevens MF Bradshaw TD . Temozolomide: mechanisms of action, repair and resistance. Curr Mol Pharmacol. (2012) 5:102–14. doi: 10.2174/1874467211205010102 22122467

[83] Chumakova A Lathia JD . Outlining involvement of stem cell program in regulation of O6-methylguanine DNA methyltransferase and development of temozolomide resistance in glioblastoma: An Editorial Highlight for ‘Transcriptional control of O(6) -methylguanine DNA methyltransferase expression and temozolomide resistance in glioblastoma’ on page 780. J Neurochem. (2018) 144:688–90. doi: 10.1111/jnc.2018.144.issue-6 29644711

[84] Perazzoli G Prados J Ortiz R Caba O Cabeza L Berdasco M . Temozolomide resistance in glioblastoma cell lines: implication of MGMT, MMR, P-glycoprotein and CD133 expression. PloS One. (2015) 10:e0140131. doi: 10.1371/journal.pone.0140131 26447477 PMC4598115

[85] Hegi ME Diserens AC Gorlia T Hamou MF de Tribolet N Weller M . MGMT gene silencing and benefit from temozolomide in glioblastoma. N Engl J Med. (2005) 352:997–1003. doi: 10.1056/NEJMoa043331 15758010

[86] Johannessen TCA Bjerkvig R . Molecular mechanisms of temozolomide resistance in glioblastoma multiforme. Expert Rev Anticancer Ther. (2012) 12:635–42. doi: 10.1586/era.12.37 22594898

[87] Jiapaer S Furuta T Tanaka S Kitabayashi T Nakada M . Potential strategies overcoming the temozolomide resistance for glioblastoma. Neurol Medico-Chirurgica. (2018) 58:405–21. doi: 10.2176/nmc.ra.2018-0141 PMC6186761 30249919

[88] Murota Y Tabu K Taga T . Cancer stem cell-associated immune microenvironment in recurrent glioblastomas. Cells. (2022) 11:2054. doi: 10.3390/cells11132054 35805138 PMC9265559

[89] Dong Z Cui H . Epigenetic modulation of metabolism in glioblastoma. Semin Cancer Biol. (2019) 57:45–51. doi: 10.1016/j.semcancer.2018.09.002 30205139

[90] Wu Q Berglund AE Etame AB . The impact of epigenetic modifications on adaptive resistance evolution in glioblastoma. Int J Mol Sci. (2021) 22:8324. doi: 10.3390/ijms22158324 34361090 PMC8347012

[91] Liu B Zhou J Wang C Chi Y Wei Q Fu Z . LncRNA SOX2OT promotes temozolomide resistance by elevating SOX2 expression via ALKBH5-mediated epigenetic regulation in glioblastoma. Cell Death Dis. (2020) 11:384. doi: 10.1038/s41419-020-2540-y 32439916 PMC7242335

[92] Rezaei O Tamizkar KH Sharifi G Taheri M Ghafouri-Fard S . Emerging role of long non-coding RNAs in the pathobiology of glioblastoma. Front Oncol. (2020) 10:625884. doi: 10.3389/fonc.2020.625884 33634032 PMC7901982

[93] Lee DH Ryu HW Won HR Kwon SH . Advances in epigenetic glioblastoma therapy. Oncotarget. (2017) 8:18577–89. doi: 10.18632/oncotarget.14612 PMC5392350 28099914

[94] Was H Krol SK Rotili D Mai A Wojtas B Kaminska B . Histone deacetylase inhibitors exert anti-tumor effects on human adherent and stem-like glioma cells. Clin Epigenet. (2019) 11:11. doi: 10.1186/s13148-018-0598-5 PMC6337817 30654849

[95] Banelli B Carra E Barbieri F Würth R Parodi F Pattarozzi A . The histone demethylase KDM5A is a key factor for the resistance to temozolomide in glioblastoma. Cell Cycle. (2015) 14:3418–29. doi: 10.1080/15384101.2015.1090063 PMC4825557 26566863

[96] Ciechomska IA Marciniak MP Jackl J Kaminska B . Pre-treatment or post-treatment of human glioma cells with BIX01294, the inhibitor of histone methyltransferase G9a, sensitizes cells to temozolomide. Front Pharmacol. (2018) 9. doi: 10.3389/fphar.2018.01271 PMC6224489 30450051

[97] Gursoy-Yuzugullu O Carman C Serafim RB Myronakis M Valente V Price BD . Epigenetic therapy with inhibitors of histone methylation suppresses DNA damage signaling and increases glioma cell radiosensitivity. Oncotarget. (2017) 8:24518–32. doi: 10.18632/oncotarget.15543 PMC5421867 28445939

[98] Lathia JD Mack SC Mulkearns-Hubert EE Valentim CLL Rich JN . Cancer stem cells in glioblastoma. Genes Dev. (2015) 29:1203–17. doi: 10.1101/gad.261982.115 PMC4495393 26109046

[99] Toh TB Lim JJ Chow EKH . Epigenetics in cancer stem cells. Mol Cancer. (2017) 16:29. doi: 10.1186/s12943-017-0596-9 28148257 PMC5286794

[100] Kondo T . Glioblastoma-initiating cell heterogeneity generated by the cell-of-origin, genetic/epigenetic mutation and microenvironment. Semin Cancer Biol. (2022) 82:176–83. doi: 10.1016/j.semcancer.2020.12.003 33453403

[101] Reddy RG Bhat UA Chakravarty S Kumar A . Advances in histone deacetylase inhibitors in targeting glioblastoma stem cells. Cancer Chemother Pharmacol. (2020) 86:165–79. doi: 10.1007/s00280-020-04109-w 32638092

[102] Mallm JP Windisch P Biran A Gal Z Schumacher S Glass R . Glioblastoma initiating cells are sensitive to histone demethylase inhibition due to epigenetic deregulation. Int J Cancer. (2020) 146:1281–92. doi: 10.1002/ijc.v146.5 31456217

[103] Bovenberg MSS Degeling MH Tannous BA . Advances in stem cell therapy against gliomas. Trends Mol Med. (2013) 19:281–91. doi: 10.1016/j.molmed.2013.03.001 23537753

[104] Qiu Z Yu Z Xu T Wang L Meng N Jin H . Novel nano-drug delivery system for brain tumor treatment. Cells. (2022) 11:3761. doi: 10.3390/cells11233761 36497021 PMC9737081

[105] Sun C Ding Y Zhou L Shi D Sun L Webster TJ . Noninvasive nanoparticle strategies for brain tumor targeting. Nanomedicine. (2017) 13:2605–21. doi: 10.1016/j.nano.2017.07.009 28756093

[106] Jena L McErlean E McCarthy H . Delivery across the blood-brain barrier: nanomedicine for glioblastoma multiforme. Drug Delivery Transl Res. (2020) 10:304–18. doi: 10.1007/s13346-019-00679-2 PMC7066289 31728942

[107] Guo J Yao Z Zhang FY Wu JZ . Application of polymer materials in targeting glioma. Anti-Cancer Agents Med Chem. (2023) 23:1284–97. doi: 10.2174/1871520623666230222142825 36815633

[108] Nan J Yang W Xie YJ Yu MH Chen Y Zhang J . Emerging nano-immunotherapeutic approaches to glioma. Small Structures. (2023) 4. doi: 10.1002/sstr.202300016

[109] Patel MM Patel BM . Crossing the blood-brain barrier: recent advances in drug delivery to the brain. CNS Drugs. (2017) 31:109–33. doi: 10.1007/s40263-016-0405-9 28101766

[110] Nie E Miao F Jin X Wu W Zhou X Zeng A . Fstl1/DIP2A/MGMT signaling pathway plays important roles in temozolomide resistance in glioblastoma. Oncogene. (2019) 38:2706–21. doi: 10.1038/s41388-018-0596-2 PMC6484760 30542120

[111] Wang L Tang S Yu Y Lv Y Wang A Yan X . Intranasal delivery of temozolomide-conjugated gold nanoparticles functionalized with anti-ephA3 for glioblastoma targeting. Mol Pharm. (2021) 18:915–27. doi: 10.1021/acs.molpharmaceut.0c00911 33417456

[112] Wu H Zhang T Liu Q Wei M Li Y Ma Q . Polydopamine-based loaded temozolomide nanoparticles conjugated by peptide-1 for glioblastoma chemotherapy and photothermal therapy. Front Pharmacol. (2023) 14:1081612. doi: 10.3389/fphar.2023.1081612 36744246 PMC9889548

[113] Zheng M Huang M Ma X Chen H Gao X . Harnessing exosomes for the development of brain drug delivery systems. Bioconjug Chem. (2019) 30:994–1005. doi: 10.1021/acs.bioconjchem.9b00085 30855944

[114] Khan MI Alsayed R Choudhry H Ahmad A . Exosome-mediated response to cancer therapy: modulation of epigenetic machinery. Int J Mol Sci. (2022) 23:6222. doi: 10.3390/ijms23116222 35682901 PMC9181065

[115] Zhang X Liu C Pei Y Song W Zhang S . Preparation of a novel raman probe and its application in the detection of circulating tumor cells and exosomes. ACS Appl Mater Interfaces. (2019) 11:28671–80. doi: 10.1021/acsami.9b09465 31318195

[116] Lee JR Kyung JW Kumar H Kwon SP Song SY Han IB . Targeted delivery of mesenchymal stem cell-derived nanovesicles for spinal cord injury treatment. Int J Mol Sci. (2020) 21:4185. doi: 10.3390/ijms21114185 32545361 PMC7312698

[117] Shaikh S Rehman FU Du T Jiang H Yin L Wang X . Real-time multimodal bioimaging of cancer cells and exosomes through biosynthesized iridium and iron nanoclusters. ACS Appl Mater Interfaces. (2018) 10:26056–63. doi: 10.1021/acsami.8b08975 30011179

[118] Cui J Xu Y Tu H Zhao H Wang H Di L . Gather wisdom to overcome barriers: Well-designed nano-drug delivery systems for treating gliomas. Acta Pharm Sin B. (2022) 12:1100–25. doi: 10.1016/j.apsb.2021.08.013 PMC9069319 35530155

[119] da Silva BA Nazarkovsky M Padilla-Chavarría HI Mendivelso EAC Mello HL Nogueira CSC . Novel scintillating nanoparticles for potential application in photodynamic cancer therapy. Pharmaceutics. (2022) 14:2258. doi: 10.3390/pharmaceutics14112258 36365077 PMC9697386

[120] Vedunova M Turubanova V Vershinina O Savyuk M Efimova I Mishchenko T . DC vaccines loaded with glioma cells killed by photodynamic therapy induce Th17 anti-tumor immunity and provide a four-gene signature for glioma prognosis. Cell Death Dis. (2022) 13:1062. doi: 10.1038/s41419-022-05514-0 36539408 PMC9767932

[121] He X Luo Y Li Y Pan Y Guo D He L . D-type neuropeptide decorated AIEgen/RENP hybrid nanoprobes with light-driven ROS generation ability for NIR-II fluorescence imaging-guided through-skull photodynamic therapy of gliomas. Aggregate. (2024), 185–95. doi: 10.1002/agt2.v5.1

[122] Agostinis P Berg K Cengel KA Foster TH Girotti AW Gollnick SO . Photodynamic therapy of cancer: an update. CA Cancer J Clin. (2011) 61:250–81. doi: 10.3322/caac.20114 PMC3209659 21617154

[123] Cramer SW Chen CC . Photodynamic therapy for the treatment of glioblastoma. Front Surg. (2019) 6:81. doi: 10.3389/fsurg.2019.00081 32039232 PMC6985206

[124] Hirschberg H Berg K Peng Q . Photodynamic therapy mediated immune therapy of brain tumors. Neuroimmunol Neuroinflamm. (2018) 5:27. doi: 10.20517/2347-8659.2018.31 30221185 PMC6138455

[125] Cheng H Fan G Fan J Yuan P Deng F Qiu X . Epigenetics-inspired photosensitizer modification for plasma membrane-targeted photodynamic tumor therapy. Biomaterials. (2019) 224:119497. doi: 10.1016/j.biomaterials.2019.119497 31541935

[126] Halaburková A Jendzelovsky R Koval J Herceg Z Fedorocko P Ghantous A . Histone deacetylase inhibitors potentiate photodynamic therapy in colon cancer cells marked by chromatin-mediated epigenetic regulation of CDKN1A. Clin Epigenet. (2017) 9:62. doi: 10.1186/s13148-017-0359-x PMC5465463 28603560

[127] Ding F Liu J Ai K Xu C Mao X Liu Z . Simultaneous activation of pyroptosis and cGAS-STING pathway with epigenetic/photodynamic nanotheranostic for enhanced tumor photoimmunotherapy. Adv Mater. (2024) 36:e2306419. doi: 10.1002/adma.202306419 37796042

[128] Pan Y Zhu Y Xu C Pan C Shi Y Zou J . Biomimetic yolk-shell nanocatalysts for activatable dual-modal-image-guided triple-augmented chemodynamic therapy of cancer. ACS Nano. (2022) 16:19038–52. doi: 10.1021/acsnano.2c08077 36315056

[129] Rong L Li N Zhang Z . Emerging therapies for glioblastoma: current state and future directions. J Exp Clin Cancer Res. (2022) 41:142. doi: 10.1186/s13046-022-02349-7 35428347 PMC9013078

[130] Xiong S Qin B Liu C Pan Y . Editorial: Immunosuppression mechanisms and immunotherapy strategies in glioblastoma. Front Cell Neurosci. (2024) 18:1411330. doi: 10.3389/fncel.2024.1411330 38725447 PMC11080981

[131] Pan Y Cheng J Zhu Y Zhang J Fan W Chen X . Immunological nanomaterials to combat cancer metastasis. Chem Soc Rev. (2024) 53:6399–444. doi: 10.1039/D2CS00968D 38745455

[132] Li B Severson E Pignon JC Zhao H Li T Novak J . Comprehensive analyses of tumor immunity: implications for cancer immunotherapy. Genome Biol. (2016) 17:174. doi: 10.1186/s13059-016-1028-7 27549193 PMC4993001

[133] Gangoso E Southgate B Bradley L Rus S Galvez-Cancino F McGivern N . Glioblastomas acquire myeloid-affiliated transcriptional programs via epigenetic immunoediting to elicit immune evasion. Cell. (2021) 184:2454–70.e26. doi: 10.1016/j.cell.2021.03.023 33857425 PMC8099351

[134] Chongsathidkiet P Jackson C Koyama S Loebel F Cui X Farber SH . Sequestration of T cells in bone marrow in the setting of glioblastoma and other intracranial tumors. Nat Med. (2018) 24:1459–68. doi: 10.1038/s41591-018-0135-2 PMC6129206 30104766

[135] Eder K Kalman B . The dynamics of interactions among immune and glioblastoma cells. Neuromol Med. (2015) 17:335–52. doi: 10.1007/s12017-015-8362-x 26224516

[136] Kanwore K Kanwore K Adzika GK Abiola AA Guo X Kambey PA . Cancer metabolism: the role of immune cells epigenetic alteration in tumorigenesis, progression, and metastasis of glioma. Front Immunol. (2022) 13:831636. doi: 10.3389/fimmu.2022.831636 35392088 PMC8980436

[137] Wang C Wang L Ding Y Lu X Zhang G Yang J . LncRNA structural characteristics in epigenetic regulation. Int J Mol Sci. (2017) 18:2659. doi: 10.3390/ijms18122659 29292750 PMC5751261

[138] Saxena A Carninci P . Long non-coding RNA modifies chromatin: epigenetic silencing by long non-coding RNAs. Bioessays. (2011) 33:830–9. doi: 10.1002/bies.201100084 PMC3258546 21915889

[139] Heerboth S Lapinska K Snyder N Leary M Rollinson S Sarkar S . Use of epigenetic drugs in disease: an overview. Genet Epigenet. (2014) 6:9–19. doi: 10.4137/GEG.S12270 25512710 PMC4251063

[140] Yu WD Wang H He QF Xu Y Wang XC . Long noncoding RNAs in cancer-immunity cycle. J Cell Physiol. (2018) 233:6518–23. doi: 10.1002/jcp.v233.9 PMC13482439 29574911

[141] Pan Y Zhu Y Zhang Q Zhang C Shao A Zhang J . Prognostic and predictive value of a long non-coding RNA signature in glioma: A lncRNA expression analysis. Front Oncol. (2020) 10:1057. doi: 10.3389/fonc.2020.01057 32793467 PMC7394186

[142] Ratnam NMM Sonnemann HMM Frederico SCC Chen HW Hutchinson M Dowdy T . Reversing epigenetic gene silencing to overcome immune evasion in CNS Malignancies. Front Oncol. (2021) 11. doi: 10.3389/fonc.2021.719091 PMC8320893 34336705

[143] Wang K Yang C Li H Liu X Zheng M Xuan Z . Role of the epigenetic modifier JMJD6 in tumor development and regulation of immune response. Front Immunol. (2022) 13. doi: 10.3389/fimmu.2022.859893 PMC8963961 35359945

